# Supplementary material for: Synthetic Lethality Screening Highlights Colorectal Cancer Vulnerability to Concomitant Blockade of NEDD8 and EGFR Pathways
Source: Cancers (Basel). 2021 Jul 28;13(15):3805. doi: 10.3390/cancers13153805 (PMC8345131; doi:10.3390/cancers13153805)
Supplement: Supplementary file 1 [file cancers-13-03805-s001.zip › cancers-1257497-supplementary.pdf]

cancers

MDPI

Supplementary Materials: Synthetic Lethality Screening Highlights Colorectal Cancer Vulnerability to Concomitant Blockade of NEDD8 and EGFR Pathways

Federica Invrea, Simona Punzi, Consalvo Petti, Rosalba Minelli, Michael D. Peoples, Christopher A. Bristow, Valentina Vurchio, Alessia Corrado, Alberto Bragoni, Caterina Marchiò, Andrea Bertotti, Livio Trusolino, Alberto Bardelli, Claudio Isella, Alessandro Carugo, Giulio F. Draetta and Enzo Medico

Supplementary Methods

Library Composition

The shRNA library targeted 200 genes (10 shRNAs per gene), which sequences were designed using a proprietary algorithm (Cellecta Inc.). shRNAs were under the control of a constitutive U6 promoter and univocally associated to a barcode cassette (BC) of 18, non-overlapping nucleotides to specifically "tag" individual constructs. The library also contained two positive (PSMA1, RPL30) and a neutral (Luciferase, LUC) controls.

shRNA Library Transduction and in vitro Screening

Concentrated lentiviral particles from the shRNAs library were produced by transfecting 293T cells, as described in the Cellecta User Manual (https://manuals.cellecta.com/rnai-pooled-lentiviral-shrna-libraries/ accessed on 1 May 2020), and were independently added in triplicate to CRC cell lines used for the *in vitro* screening, together with 8 μg/mL polybrene (Sigma) for 24 hours and then standard growth medium was replaced. At 72h post transduction, the percentage of infected cells was determined through flow cytometry analysis and 3 μg/mL of puromycin was added before performing the experiments. Library infection was performed using a Multiplicity of Infection (MOI) of ~0.2 Transducing Unit/cell. Cells were then amplified for approximately twenty doublings in presence (+) or ten doublings in absence (−) of pevonedistat at a concentration that reduced growth rate rather than totally impair cell proliferation. Pevonedistat concentration, specifically chosen for each cell line, was 25 nM for CAR1, 100 nM for LIM2099 and SW403 and 200 nM for WIDR. Genomic DNA (gDNA) was extracted immediately from cells after complete puromycin selection as reference, and after cellular doublings +/− pevonedistat. At every passage, cells were manually counted and at least 4 million were re-plated to preserve the correct representation (1000× coverage) of the library. GDNA extraction, PCR assay and Next Generation Sequencing (NGS) were performed according to Cellecta User Manual.

Sequences and Data Analysis

BCs representation was measured by NGS on Illumina NextSeq and BCs were identified by aligning each sequencing read to the barcoded-libraries using the Bowtie aligner (2.0.2), and by considering only those BCs having, at most, three mismatches in each alignment. Upon quantification each shRNA barcode, data were normalized on library size calculating counts *per* millions (CPM). Accordingly, library constructs were represented with more than one CPM in more than 99.9% in the unselected cells supporting the complete representation and capture of the library. Each experiment included two control points: the basal, library infected unselected cells, the library infected cells after 20 cell doublings, and library infected cells selected with pevonedistat treatment. To ensure robust identification of shRNA construct depletion in pevonedistat selection, in each replicate we calculated for each construct the LOG_2 fold change of comparing pevonedistat selected cells and the minimum value of the controls. On such data, Lucif-

erase targeting shRNAs, as neutral controls, were employed as to define minimum threshold to call the depletion. To minimize false calling, each single shRNA targeting construct was then called depleted if ranked below the 33th percentile of the distribution in each replicate for CAR1, LIM2099 and SW403; WiDr required a 25th percentile threshold. Finally, to identify gene depletion we required a minimum of four shRNAs to be depleted in all the biological replicates.

Immunohistochemistry

After deparaffinization, endogenous peroxidase was blocked in 3% hydrogen peroxide and heating antigen retrieval was carried in citrate buffer (pH 6.0). Sections were stained with monoclonal mouse anti-human Ki67 (Dako), p21 and p27 (#2947 and #3686; Cell signaling Technology) antibodies. Immunoreactivities were revealed by incubation in DAB chromogen (Dako). Slides were counterstained in Mayer's hematoxylin. Images were acquired by Olympus AX70 up-right microscope (objective UPLFLN 10×) and processed by using Cell Sens Science Imaging Software (Olympus corporation, Japan). Images are presented at 10X magnification (scale bar 200 μm). The markers' quantification (Ki67, p21 and p27) was calculated on more representative areas of the tumors for at least 15 microscopic fields in different sections and expressed as percentage (mean ± SD).

Supplementary Figures

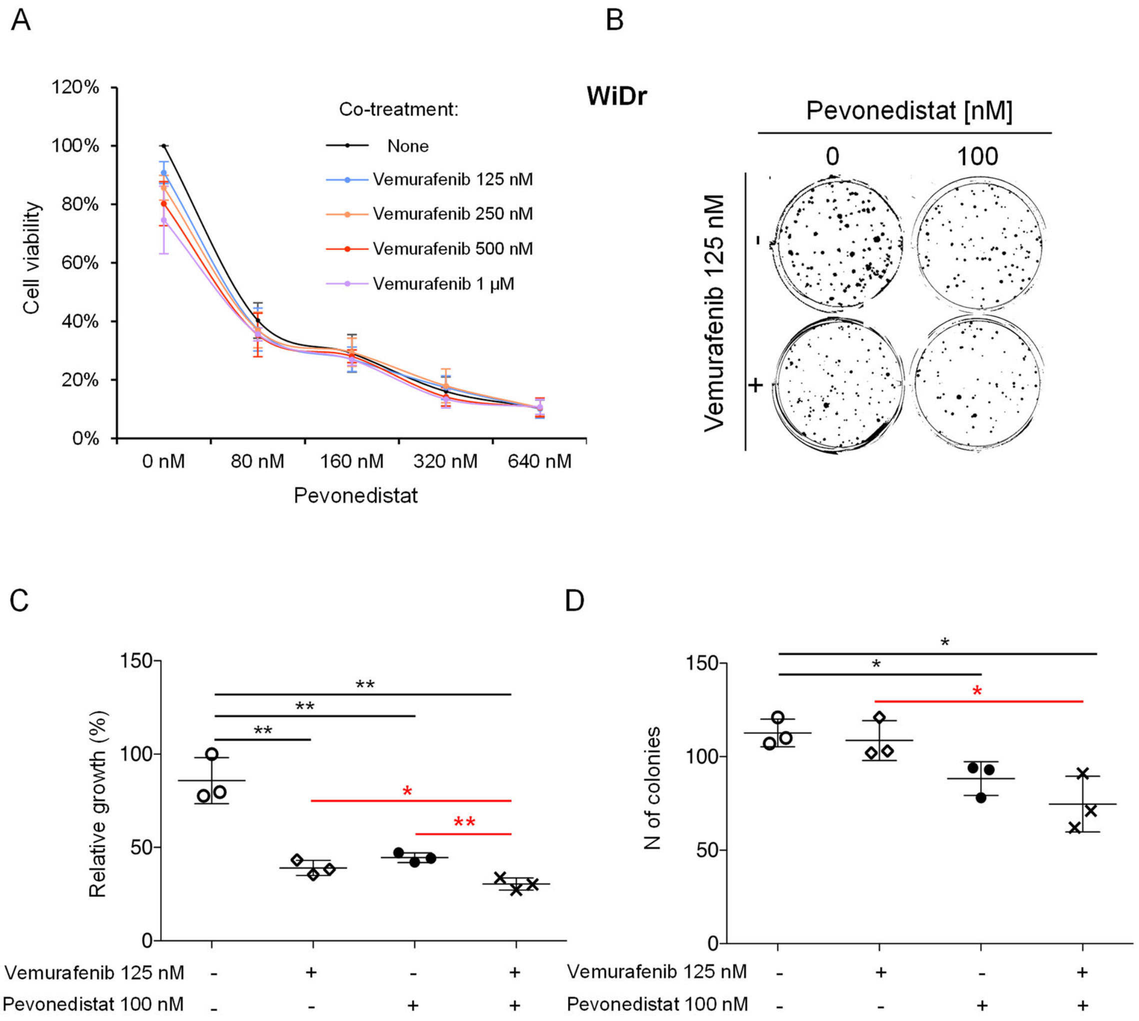

Figure S1. Evaluation of pevonedistat and vemurafenib sensitivity on *BRAF*-mutant WiDr cells. (**A**) Short-term dose–response proliferation assay on WiDr treated with increasing concentrations of pevonedistat combined with different doses of vemurafenib. At 96h the percentage of cell viability was estimated and normalized to the untreated cells. Data are expressed as average ± SD of four technical replicates. (**B**) Colonies formation of WiDr *BRAF*-mutant cells was evaluated in response to 100 nM of pevonedistat after 17 days of treatment in presence and absence of BRAF inhibitor vemurafenib (125 nM). Cells were fixed by using Cristal Violet. (**C**) WiDr colonies relative growth was quantified by ImageJ analysis in triplicate and expressed as relative growth in percentage. Significant differences among groups were evaluated by Student *t* test (*: $p < 0.05$; **: $p < 0.01$). (**D**) Number of WiDr colonies was quantified by ImageJ analysis in triplicate. Significant differences among groups were evaluated by Student *t* test (*: $p < 0.05$).

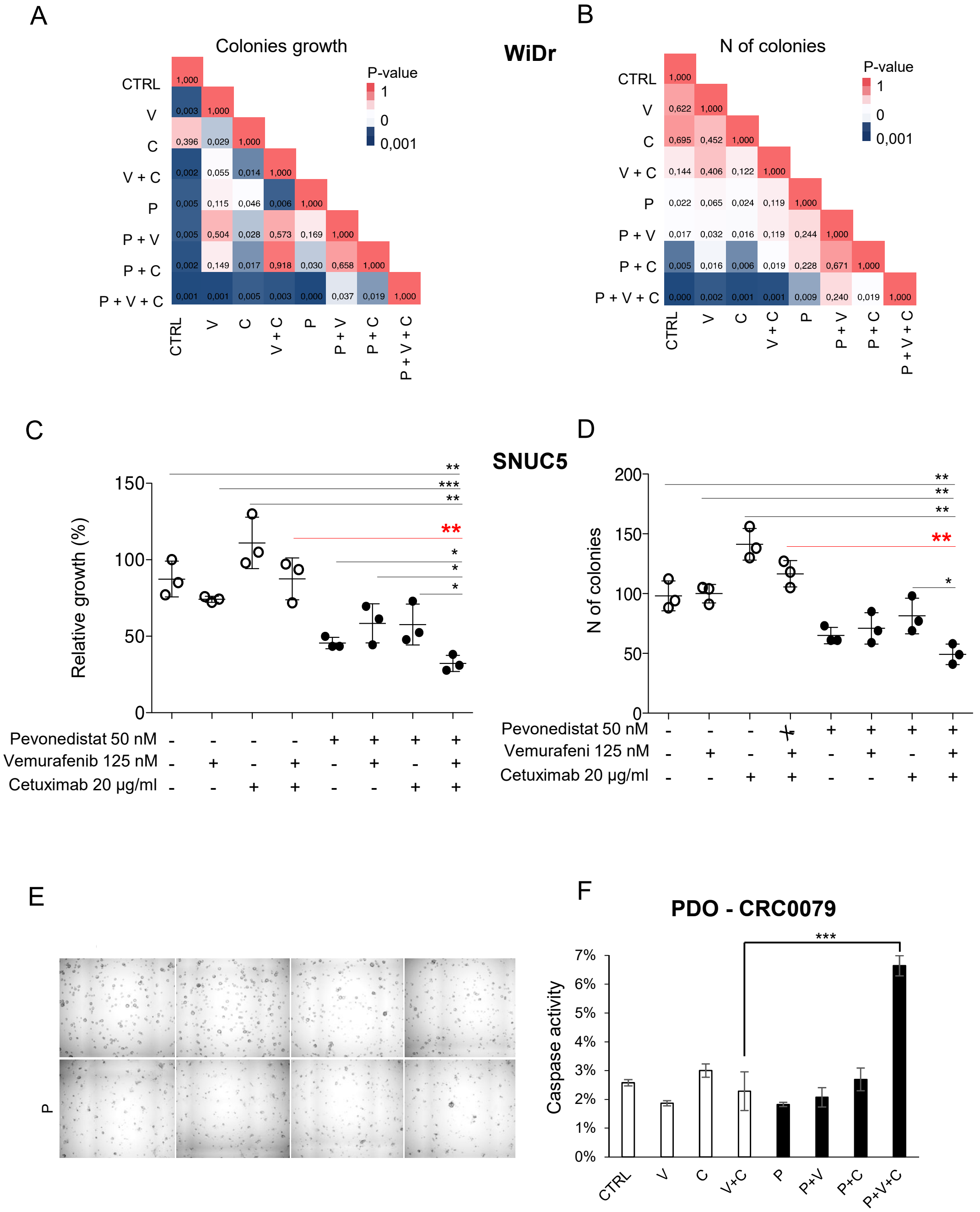

Figure S2. Effect of pevonedistat combined with EGFR inhibition on colony formation in *BRAF*-mutant CRC cell lines and organoids. A–B. WiDr cells were used to perfom a colony formation assay and response to pevonedistat (100 nM), cetuximab (20 µg/ml), vemurafenib (125 nM) alone or in combination was evaluated. After 17 days cells were fixed by crystal violet. Relative growth of WiDr colonies (**A**) and their number (**B**) were quantified in triplicate and significant differences among groups (pevonedistat–P; vemurafenib–V; cetuximab–C) were analyzed by applying a Student *t* test as show in the heat maps. C-D. An additional *BRAF*-mutant CRC cell line SNUC5 was used to perfom a colony formation assay and response to pevonedistat (100 nM), cetuximab (20 µg/mL), vemurafenib (125 nM) alone or in combination was evaluated. After 8 days cells were fixed by crystal violet. Relative growth of SNUC5 colonies (**C**) and their number (**D**) were quantified in triplicate and significant differences among triple combination and other treatment groups (pevonedistat–P; vemurafenib–V; cetuximab–C) were analyzed by applying a Student *t* test (*: $p < 0.05$; **: $p < 0.01$; ***: $p < 0.001$). (**E**) *BRAF*-mutant Patient-Derived Organoid (PDO – CRC0079) was treated for 96h with vemurafenib (125 nM), cetuximab (20 µg/ml) and increasing doses of pevonedistat (150 - 300 - 500 nM). Representative images of three technical replicates at 500 nM pevonedistat dose are reported. (**F**) Caspase-3/7 activity was quantified by Caspase Glo Assay after 24h treatment and expressed as ratio of proliferation. Significant differences among V+C and V+C+P groups were calculated by applying Student *t* test (***: $p < 0.001$).

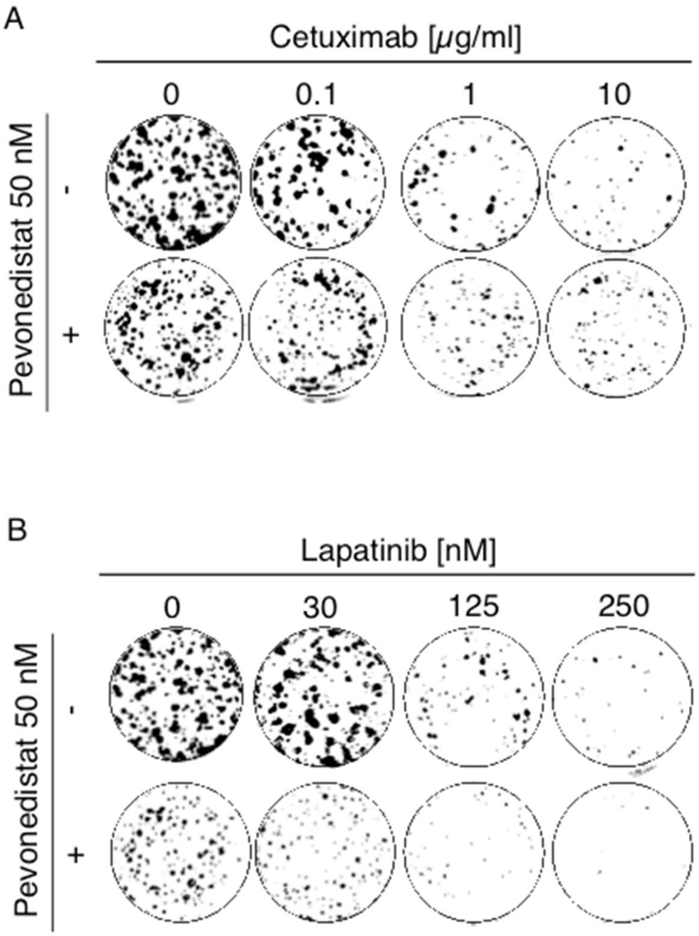

Figure S3. Evaluation of pevonedistat sensitivity combined with anti-EGFR inhibitors on *RAS*/*RAF* WT HCA7 cells. (**A**–**B**) Colony formation of HCA7 cells was evaluated in response to pevonedistat 50 nM, increasing concentrations of cetuximab (**A**) or lapatinib (**B**) and combinatorial treatments of both. After 13 days cells were fixed by crystal violet. Representative images of three technical replicates are reported.

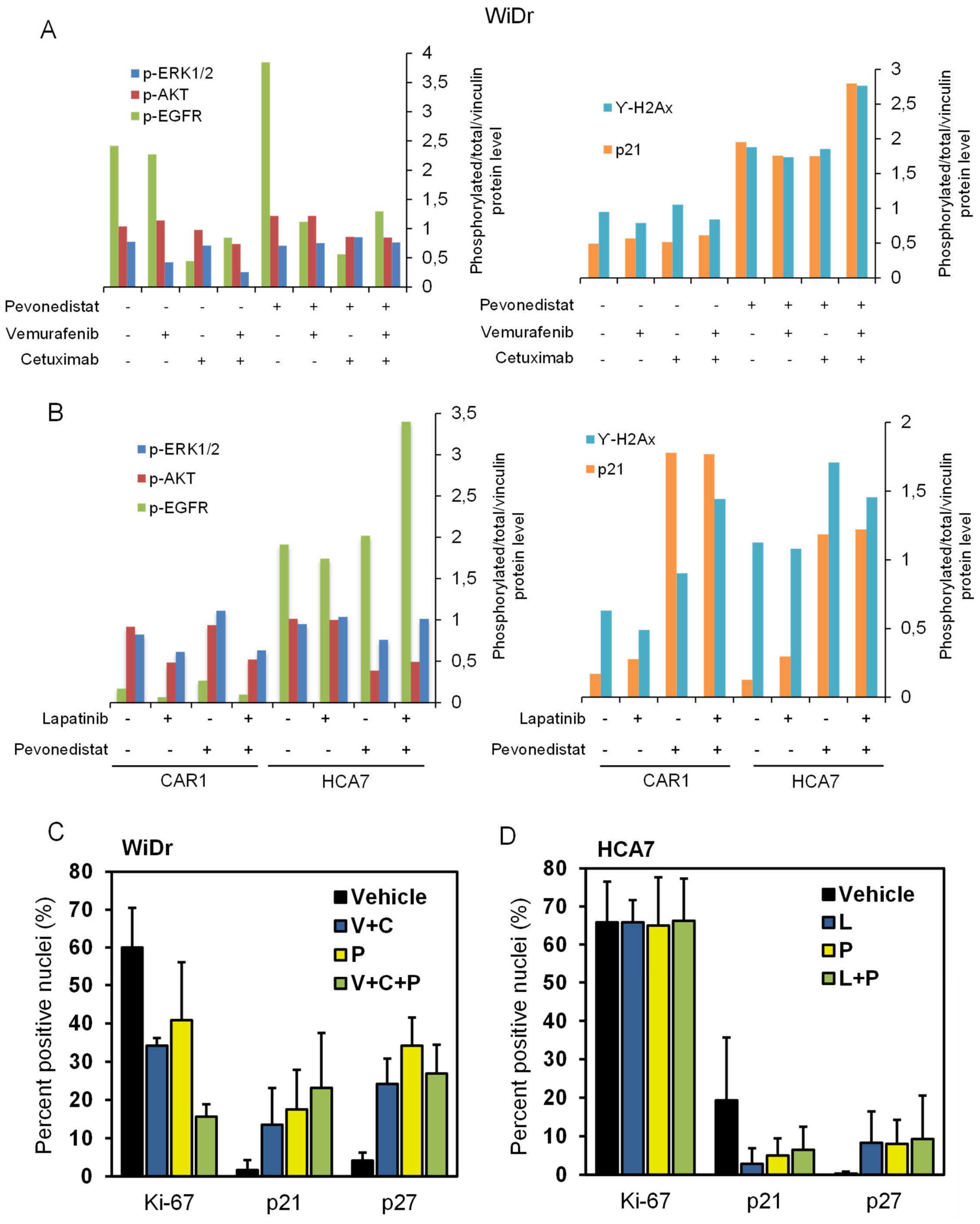

Figure S4. Quantification of western blot and immunohistochemistry on *BRAF*-mutant and *RAS*/*RAF WT* cells (see Main Figure 5). (**A**) Protein quantification from western blot on WiDr lysates (referred to Main Figure 5A) was performed by using ImageJ software. The level of the phosphorylated forms was expressed as ratio to the respective total form, after normalization of each sample loading by using vinculin. (**B**) Protein quantification from western blot on CAR1 and HCA7 lysates (referred to Main Figure 5C,D) was performed by using ImageJ software. The level of the phosphorylated forms was expressed as ratio to the respective total form, after normalization of each sample loading by using vinculin. (**C**) The quantification of immunohistochemistry markers Ki67, p21 and p27 on WiDr xenografts (referred to Main Figure 5B) was calculated on more representative areas of the tumors for at least 15 microscopic fields in different sections and expressed as percentage (mean ± SD). (**D**) The quantification of immunohistochemistry markers Ki67, p21 and p27 on HCA7 xenografts (referred to Main Figure 5E) was calculated on more representative areas of the tumors for at least 15 microscopic fields in different sections and expressed as percentage (mean ± SD).

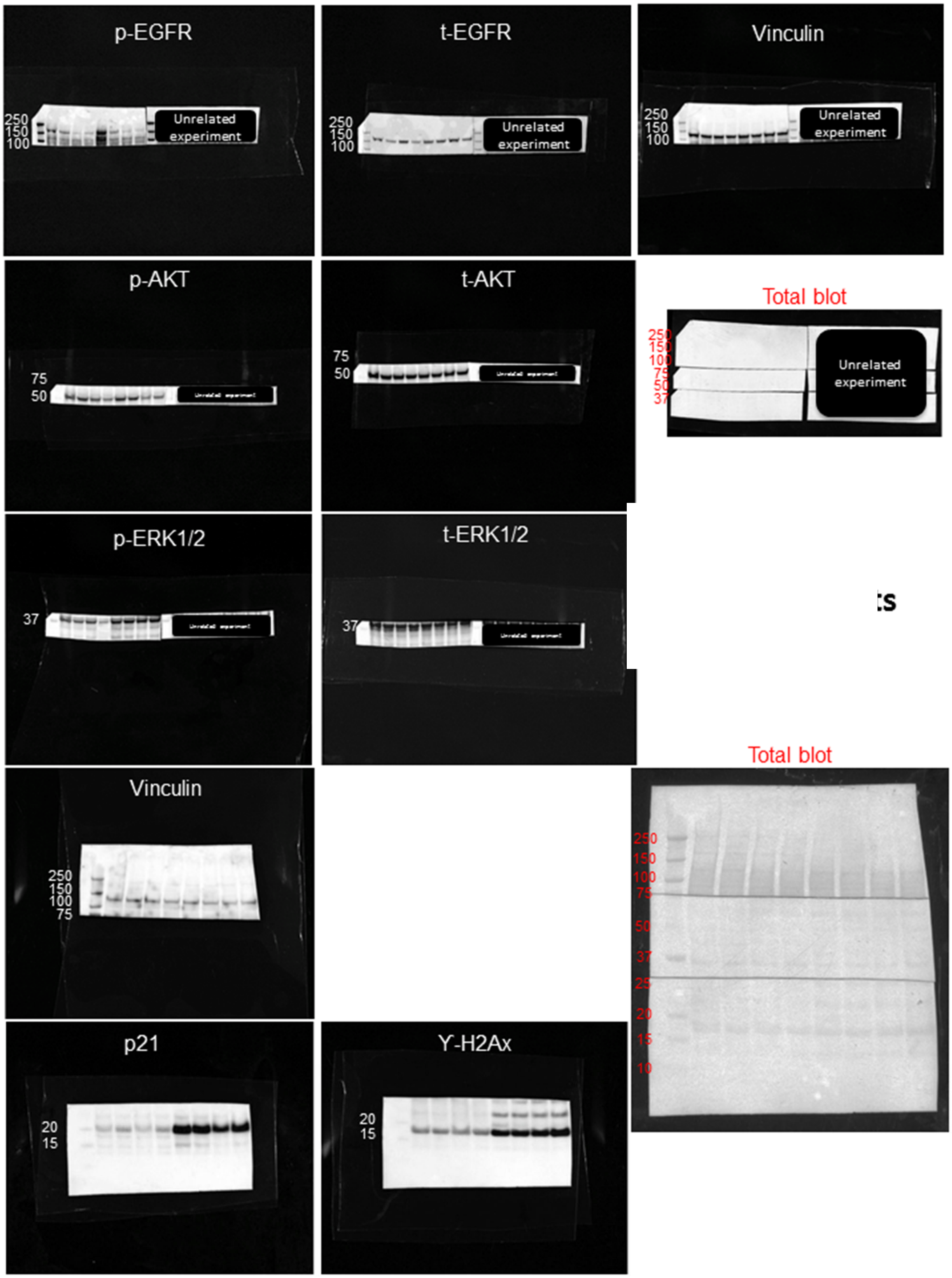

p-EGFR
250
150
100
Unrelated experiment
t-EGFR
250
150
100
Unrelated experiment
Vinculin
250
150
100
Unrelated experiment
p-AKT
75
50
t-AKT
75
50
Total blot
250
150
100
75
50
37
Unrelated experiment
p-ERK1/2
37
t-ERK1/2
37
Vinculin
250
150
100
75
Total blot
250
150
100
75
50
37
25
20
15
10
p21
20
15
Y-H2Ax
20
15

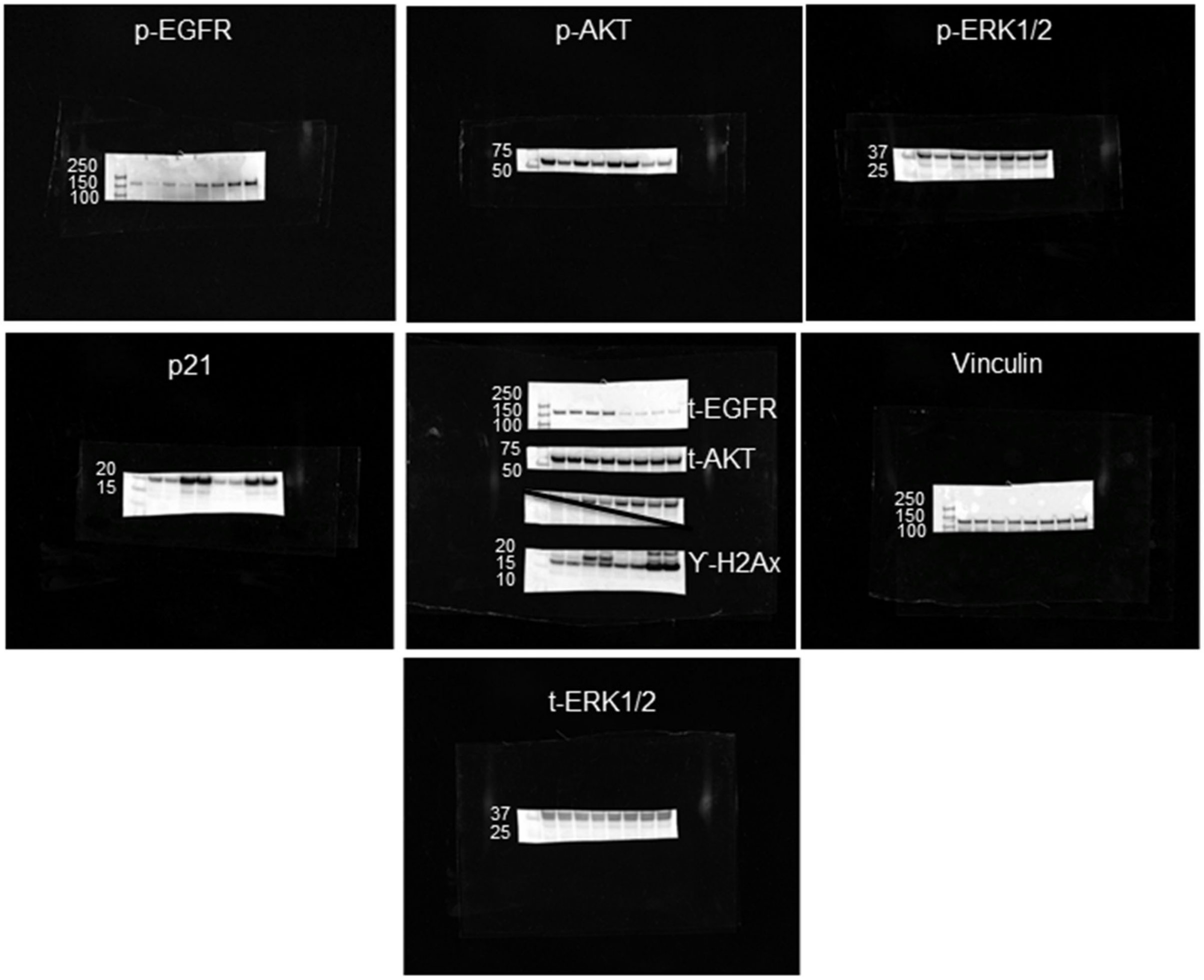

Figure S5. The uncropped Western blots.

Table S1. shRNA library details and screening candidate hit genes. (**A**) Genes composing the shRNA library, Ensembl annotation, drugs available targeting each gene and corresponding pathway/signaling or function are listed. (**B**) shRNAs targeting each gene are reported. For each cell line sequenced, LOG_2 ratio of each replicate was calculated; call for depletion in all three replicates; gene depletion call. (**C**) Expression levels expressed as TMM of each candidate gene hit in the CRC cell lines used for the synthetic lethal screening.

Supplementary Table S1A. Genes composing the shRNA library, Ensembl annotation, drugs available targeting each gene and corresponding pathway/signaling or function.

Target_Gene	Ensembl Gene ID	Targeting Drug(s)	Pathway/Signaling/Function
ABL1	ENSG00000097007	adenosine-triphosphate<br>bafetinib<br>bosutinib<br>dasatinib<br>flumatinib<br>GNF-2<br>GZD824<br>imatinib<br>KW-2449<br>LDN-212854<br>metatinib<br>nilotinib<br>ponatinib<br>PP-121<br>PP-2<br>rebastinib<br>regorafenib<br>saracatinib	RAS-ERK signaling pathway
ABL2	ENSG00000143322	adenosine-triphosphate<br>dasatinib	RAS-ERK signaling pathway
AKT1	ENSG00000142208	A-674563<br>adenosine-triphosphate<br>arsenic-trioxide<br>AT13148<br>AZD5363<br>bisindolylmaleimide-ix<br>BMS-536924<br>BMS-754807<br>CaMKII-IN-1<br>canertinib<br>enzastaurin<br>GDC-0068<br>GSK2110183<br>GSK690693<br>hexamethylenebisacetamide<br>KN-62<br>LY294002<br>MK-2206<br>PD-98059<br>perifosine<br>SB-202190<br>SB-203580<br>TIC10	HGF-MET-PI3K signaling pathway

		triciribine<br>U-0126<br>uprosertib	
AKT2	ENSG00000105221	ARQ-092<br>AT13148<br>AT7867<br>AZD5363<br>GDC-0068<br>GSK2110183<br>GSK690693<br>MK-2206<br>PHT-427<br>triciribine<br>uprosertib	HGF-MET-PI3K signaling pathway
AKT3	ENSG00000117020	AT13148<br>AZD5363<br>GDC-0068<br>GSK2110183<br>GSK690693<br>MK-2206<br>triciribine<br>uprosertib	HGF-MET-PI3K signaling pathway
ALK	ENSG00000171094	adenosine-triphosphate<br>alectinib<br>AP26113<br>ASP3026<br>AZD3463<br>brigatinib<br>CEP-37440<br>ceritinib<br>crizotinib<br>entrectinib<br>GSK1838705A<br>lorlatinib<br>NVP-TAE684<br>PF-06463922	RAS-ERK signaling pathway
AR	ENSG00000169083	andarine<br>APC-100<br>apigenin<br>ARN-509<br>ASC-J9<br>AZD3514<br>bicalutamide<br>bisphenol-a<br>CB-03-01<br>cortodoxone<br>cyproterone<br>cyproterone-acetate<br>danazol<br>dehydroepiandrosterone-sulfate<br>dihydrotestosterone	Androgen receptor signaling pathway

		dromostanolone-propionate<br>drospirenone<br>enzalutamide<br>epitiostanol<br>eugenol<br>fludrocortisone-acetate<br>flufenamic-acid<br>fluoxymesterone<br>flutamide<br>galeterone<br>gestrinone<br>GLPG0492<br>GLPG0492-R-enantiomer<br>kaempferol<br>ketoconazole<br>levonorgestrel<br>LXR-623<br>LY2452473<br>mifepristone<br>MK-0773<br>nandrolone<br>nandrolone-decanoate<br>nilutamide<br>ODM-201<br>ostarine<br>oxandrolone<br>oxymetholone<br>PS178990<br>pyrvinium-pamoate<br>RU-58841<br>spironolactone<br>stanozolol<br>testosterone<br>testosterone-enanthate<br>testosterone-propionate<br>testosterone-undecanoate<br>17-alpha-methyltestosterone<br>17-hydroxyprogesterone-caproate<br>2-hydroxyflutamide	
ATM	ENSG00000149311	caffeine<br>CGK-733<br>KU-55933<br>KU-60019<br>VE-822	ATM-TP53 pathway
ATR	ENSG00000175054	AZD6738<br>AZ20<br>CGK-733<br>NVP-BEZ235<br>schisandrin-b<br>VE-821	ATR-TP53 pathway

		VE-822	
AURKA	ENSG00000087586	alisertib<br>AMG900<br>AT-9283<br>aurora-a-inhibitor-i<br>barasertib<br>BI-847325<br>CCT129202<br>CCT137690<br>CYC116<br>danusertib<br>ENMD-2076<br>GSK1070916<br>JNJ-7706621<br>KW-2449<br>MK-5108<br>MK-8745<br>MLN-8054<br>orantinib<br>PF-03814735<br>PHA-680632<br>SNS-314<br>tozasertib<br>ZM-447439	PI3K-AKT-MTOR pathway
AURKB	ENSG00000178999	AMG900<br>AT-9283<br>barasertib<br>barasertib-HQPA<br>BI-847325<br>CCT129202<br>CCT137690<br>CGP-57380<br>CYC116<br>danusertib<br>GSK1070916<br>hesperadin<br>JNJ-7706621<br>KW-2449<br>MK-5108<br>orantinib<br>PF-03814735<br>PHA-680632<br>reversine<br>SNS-314<br>TAK-901<br>tozasertib<br>ZM-447439	PI3K-AKT-MTOR pathway
AURKC	ENSG00000105146	AMG900<br>BI-847325<br>CCT129202<br>CCT137690	PI3K-AKT-MTOR pathway

		danusertib<br>GSK1070916<br>MK-5108<br>PHA-680632<br>SNS-314<br>tozasertib	
AXL	ENSG00000167601	BMS-777607<br>LDC1267<br>MGCD-265<br>R-428<br>TP-0903	RAS-ERK signaling pathway
BCL2	ENSG00000171791	ABT-737<br>docetaxel<br>edaravone<br>gambogic-acid<br>gossypol<br>HA-14-1<br>HA14-1<br>ibuprofen-(s)<br>navitoclax<br>obatoclax<br>oridonin<br>paclitaxel<br>rasagiline<br>TW-37<br>venetoclax	BCL2 Family
BCR	ENSG00000186716	bafetinib<br>bosutinib<br>GNF-2<br>GZD824<br>metatinib<br>ponatinib<br>rebastinib	RAS-ERK signaling pathway
BIRC5	ENSG00000089685	terameprocol<br>YM-155	Wnt signaling pathway
BLK	ENSG00000136573	dasatinib<br>ibrutinib	SRC tyrosin kinase signaling
BMX	ENSG00000102010	ibrutinib	SRC tyrosin kinase signaling
BRAF	ENSG00000157764	AZ-628<br>CEP-32496<br>dabrafenib<br>GDC-0879<br>LGX818<br>LY3009120<br>PLX-4720<br>PLX8394<br>RAF265<br>regorafenib<br>Ro-5126766<br>SB-590885<br>sorafenib<br>TAK-632	BRAF to ERK signaling pathway

		vemurafenib	
		ZM-336372	
BRD4	ENSG00000141867	ARV-825	Bromodomain and extra terminal domain family
		BI-2536	
		CPI-0610	
		CPI-203	
		I-BET-762	
		I-BET151	
		JQ1-(+)	
		LY303511	
		OTX015	
		PFI-1	
		TG-101348	
BTK	ENSG00000010671	acalabrutinib	NFKB pathway
		AVL-292	
		CNX-774	
		GDC-0834	
		ibrutinib	
		LFM-A13	
		olmutinib	
		ONO-4059	
		terreic-acid-(-)	
CASP3	ENSG00000164305	amygdalin	TNF signaling pathway
		AZ-10417808	
		DBeQ	
		minocycline	
		NQDI-1	
		PAC-1	
		PETCM	
		sanguinarium-chloride	
CCR5	ENSG00000160791	adaptavir	NFKB/PI3K/MAPK pathways
		maraviroc	
		TAK-220	
		vicriviroc	
CD274	ENSG00000120217	PD1-PDL-inhibitor-1	PDL1-PD1 pathway
CDK1	ENSG00000170312	alvocidib	Cell cycle regulator
		aminopurvalanol-a	
		AT-7519	
		BMS-265246	
		CDK1-5-inhibitor	
		CHIR-99021	
		dinaciclib	
		indirubin	
		indirubin-3-monoxime	
		JNJ-7706621	
		kenpaullone	
		olomoucine	
		PF-573228	
		PHA-767491	
		PHA-793887	
		purvalanol-a	

		P276-00<br>RGB-286638<br>R547<br>SU9516<br>terameprocol<br>TG-02<br>1-azakenpaullone<br>8-hydroxy-DPAT	
CDK2	ENSG00000123374	AG-555<br>alvocidib<br>aminopurvalanol-a<br>AT-7519<br>BMS-265246<br>BMS-387032<br>BMS-536924<br>bosutinib<br>BX-795<br>BX-912<br>dinaciclib<br>indirubin-3-monoxime<br>JNJ-7706621<br>NSC-625987<br>NU6027<br>olomoucine<br>PF-573228<br>PHA-793887<br>PHA-848125<br>purvalanol-a<br>purvalanol-b<br>RGB-286638<br>roscovitine<br>RX-3117<br>ryuvidine<br>R547<br>SB-216763<br>SCH-900776<br>SU9516<br>TG-02	Cell cycle regulator
CDK4	ENSG00000135446	alvocidib<br>AMG-925<br>arcyriaflavin-a<br>AT-7519<br>LY2835219<br>NSC-625987<br>ON123300<br>palbociclib<br>PHA-793887<br>PHA-848125<br>purvalanol-a<br>purvalanol-b<br>P276-00<br>RGB-286638	Cell cycle regulator

		ribociclib<br>ryuvidine<br>R547<br>SU9516	
CDK6	ENSG00000105810	alvocidib<br>AMG-925<br>aminopurvalanol-a<br>apigenin<br>AT-7519<br>chrysin<br>fisetin<br>LY2835219<br>palbociclib<br>RGB-286638<br>ribociclib	Cell cycle regulator
CDK7	ENSG00000134058	alvocidib<br>BMS-387032<br>BS-181<br>PF-573228<br>PHA-793887<br>PHA-848125<br>RGB-286638<br>R547<br>TG-02<br>THZ1<br>THZ1-R<br>THZ2	Cell cycle regulator
CDK9	ENSG00000136807	alvocidib<br>AT-7519<br>BMS-387032<br>CDK9-IN-6<br>dinaciclib<br>LY2857785<br>PHA-793887<br>P276-00<br>RGB-286638<br>roscovitine<br>TG-02	Cell cycle regulator
CHEK1	ENSG00000149554	AZD7762<br>BX-795<br>BX-912<br>CHIR-124<br>KN-62<br>LY2603618<br>LY2606368<br>LY294002<br>PD-98059<br>PF-477736<br>SB-202190<br>SB-203580<br>SB-218078	ATR pathway

		SCH-900776<br>U-0126<br>7-hydroxystaurosporine<br>8-hydroxy-DPAT	
CHEK2	ENSG00000183765	AZD7762<br>PF-477736<br>8-hydroxy-DPAT	ATM pathway
CREBBP	ENSG00000005339	SGC-CBP30	RAS-ERK pathway
CTNNB1	ENSG00000168036	ICG-001<br>PNU-74654<br>urea	Wnt signaling pathway
DHFR	ENSG00000228716	aminopterin<br>chlorproguanil<br>coenzyme-i<br>dapsone<br>methotrexate<br>pemetrexed<br>pralatrexate<br>proguanil<br>pyrimethamine<br>succinylsulfathiazole<br>sulfameter<br>trimethoprim	Reductase family
DOT1L	ENSG00000104885	EPZ-5676<br>EPZ004777	Histone methyltransferase
DRD2	ENSG00000149295	acepromazine<br>acetophenazine<br>adoprazine<br>afalanine<br>AJ76-(+)<br>alizapride<br>amantadine<br>amisulpride<br>amoxapine<br>aniracetam<br>aripiprazole<br>asenapine<br>azaperone<br>benperidol<br>blonanserin<br>BP-897<br>brexpiprazole<br>bromocriptine<br>bromopride<br>bromperidol<br>buspirone<br>butaclamol<br>cabergoline<br>cariprazine<br>carmoxirole<br>chlorpromazine	Dopamine receptor family

chlorprothixene
clebopride
clorotepine
clozapine
deoxyepinephrine
desipramine
dihydrexidine
dihydroergocristine
dihydroergotamine
domperidone
dopamine
doxepin
droperidol
ergotamine
eticlopride
flupentixol
fluphenazine
fluphenazine-decanoate
fluspirilene
haloperidol
haloperidol-decanoate
iloperidone
imipramine
itopride
JNJ-37822681
L-693403
L-745870
levodopa
levomepromazine
levosulpiride
lisuride
loxapine
lumateperone
lurasidone
maprotiline
melperone
memantine
mesoridazine
metoclopramide
mianserin
minaprine
mirtazapine
molindone
nafadotride
nemonapride
nomifensine
nortriptyline
olanzapine
paliperidone
pardoprunox
PD-128907

PD-168077
pergolide
perospirone
perphenazine
pimavanserin
pimozide
pipamperone
pipotiazine-palmitate
piribedil
pramipexole
preclamol
prochlorperazine
promazine
promethazine
quetiapine
quinagolide
quinelorane
quinpirol-(-)
r(-)-apomorphine
raclopride
remoxipride
risperidone
ropinirole
rotigotine
rotundine
SCH-202676
sertindole
SNAP-94847
spiperone
sumanirole
talipexole
terguride
tetrabenazine
thiethylperazine
thioproperazine
thioridazine
thiothixene
tiapride
trifluoperazine
triflupromazine
trimethobenzamide
trimipramine
UH-232-(+)
vilazodone
yohimbine
ziprasidone
zotepine
zuclopenthixol
2-CMDO
3'-fluorobenzylspiperone
7-hydroxy-DPAT

		8-hydroxy-PIPAT	
EGFR	ENSG00000146648	AEE788<br>afatinib<br>AG-490<br>alvocidib<br>AP26113<br>AST-1306<br>AV-412<br>AZD3759<br>AZD8931<br>AZ5104<br>BIBU-1361<br>BIBX-1382<br>BMS-599626<br>BMS-690514<br>brigatinib<br>canertinib<br>CGP-52411<br>CGP-53353<br>chrysophanic-acid<br>CNX-2006<br>CUDC-101<br>dacomitinib<br>dovitinib<br>EGF816<br>erbstatin-analog<br>erlotinib<br>gefitinib<br>GW-583340<br>HKI-357<br>icotinib<br>lapatinib<br>lidocaine<br>mubritinib<br>neratinib<br>olmutinib<br>orantinib<br>OSI-420<br>osimertinib<br>PD-153035<br>PD-158780<br>PD-168393<br>pelitinib<br>PKI-166<br>poziotinib<br>PP-121<br>rociletinib<br>tyrphostin-AG-1478<br>tyrphostin-AG-18<br>tyrphostin-AG-494<br>tyrphostin-AG-835<br>tyrphostin-AG-99<br>vandetanib	EGFR-RAS-ERK signaling pathway

		vatalanib<br>WHI-P154<br>WZ-3146<br>WZ-4002<br>WZ8040<br>XL-647<br>ZM-306416	
EHMT2	ENSG00000204371	A-366<br>BIX-01294<br>BRD4770<br>UNC0224<br>UNC0321<br>UNC0631<br>UNC0638<br>UNC0642<br>UNC0646<br>UNC0737	Histone Lysine Methyltransferase
EIF4E	ENSG00000151247	4EGI-1<br>4E1RCat	RAS-ERK pathway
EPHA2	ENSG00000142627	dasatinib<br>regorafenib<br>vandetanib	Tyrosine-Protein Kinase Receptor family
ERBB2	ENSG00000141736	AEE788<br>afatinib<br>ARRY-334543<br>AST-1306<br>AV-412<br>AZD8931<br>BIBX-1382<br>BMS-536924<br>BMS-599626<br>BMS-690514<br>canertinib<br>CP-724714<br>CUDC-101<br>dacomitinib<br>FERb-033<br>GW-583340<br>lapatinib<br>mubritinib<br>neratinib<br>PD-168393<br>poziotinib<br>TAK-285<br>tucatinib<br>tyrphostin-AG-825<br>tyrphostin-AG-879<br>vandetanib<br>WAY-200070<br>WZ-4002<br>XL-647	EGF receptor family of receptor tyrosine kinas-es
ESR1	ENSG00000091831	afimoxifene	Oestrogen receptor signaling

allylestrenol
AZD9496
bazedoxifene
benfluralin
bisphenol-a
bithionol
chlorotrianisene
clomifene
danazol
dehydroepiandrosterone
dehydroepiandrosterone-sulfate
desogestrel
dienestrol
diethylstilbestrol
droloxifene
endoxifen
equol
erteberel
estetrol
estradiol
estradiol-acetate
estradiol-benzoate
estradiol-cypionate
estradiol-valerate
estramustine
estramustine-phosphate
estriol
estrone
estropipate
ethinyl-estradiol
ethynodiol-diacetate
etonogestrel
fluoxymesterone
fosfestrol
fulvestrant
GDC-0810
genistein
gestrinone
hexestrol
lasofoxifene
levonorgestrel
medroxyprogesterone
melatonin
mestranol
mitotane
naloxone
norgestimate
ospemifene
PPT
progesterone
promestriene

		quinestrol<br>raloxifene<br>SNG-1153<br>tamoxifen<br>tibolone<br>toremifene<br>trilostane<br>Y-134<br>ZK-164015<br>17-hydroxyprogesterone-caproate	
ESR2	ENSG00000140009	afimoxifene<br>bazedoxifene<br>bisphenol-a<br>bithionol<br>chlorotrianisene<br>C11-Acetate<br>dehydroepiandrosterone<br>dehydroepiandrosterone-sulfate<br>diarylpropionitrile<br>diethylstilbestrol<br>equol<br>erteberel<br>estradiol<br>estramustine<br>estramustine-phosphate<br>estriol<br>estrone<br>estropipate<br>ethinyl-estradiol<br>fosfestrol<br>fulvestrant<br>genistein<br>hexestrol<br>lasofoxifene<br>morin<br>naringenin<br>ospemifene<br>PHTPP<br>prinaberel<br>raloxifene<br>tamoxifen<br>trilostane<br>WAY-200070<br>Y-134<br>ZK-164015<br>17-hydroxyprogesterone-caproate	Oestrogen receptor signaling
EZH2	ENSG00000106462	CPI-169<br>CPI-360<br>EI1<br>EPZ005687	Histone-lysine N-methyltransferase

		EPZ011989<br>GSK2816126<br>GSK503<br>tazemetostat<br>UNC1999<br>3-deazaneplanocin-A	
FGFR1	ENSG00000077782	AZD4547<br>brivanib<br>CH-5183284<br>danusertib<br>dovitinib<br>erdafitinib<br>HMN-214<br>lucitanib<br>LY2874455<br>MK-2461<br>nintedanib<br>NVP-BGJ398<br>orantinib<br>pazopanib<br>PD-161570<br>PD-173074<br>ponatinib<br>regorafenib<br>RG1530<br>semaxanib<br>sorafenib<br>TG-100801	FGF signaling pathway
FGFR2	ENSG00000066468	AEE788<br>AZD4547<br>CH-5183284<br>dovitinib<br>erdafitinib<br>LY2874455<br>MK-2461<br>nintedanib<br>NVP-BGJ398<br>orantinib<br>PD-173074<br>ponatinib<br>regorafenib<br>RG1530<br>thalidomide	FGF signaling pathway
FGFR3	ENSG00000068078	AEE788<br>AZD4547<br>brivanib-alaninate<br>CH-5183284<br>dovitinib<br>erdafitinib<br>LY2874455<br>masitinib<br>MK-2461	FGF signaling pathway

		nintedanib<br>NVP-BGJ398<br>pazopanib<br>PD-173074<br>ponatinib	
FGFR4	ENSG00000160867	AZD4547<br>BLU9931<br>erdafitinib<br>LY2874455<br>nintedanib<br>NVP-BGJ398<br>PD-173074<br>ponatinib	FGF signaling pathway
FGR	ENSG00000000938	dasatinib<br>GDC-0980<br>PRT062607<br>rebastinib	FGF signaling pathway
FLT1	ENSG00000102755	axitinib<br>brivanib<br>cediranib<br>dovitinib<br>foretinib<br>HMN-214<br>KRN-633<br>linifanib<br>lucitanib<br>MK-2461<br>motesanib<br>nintedanib<br>pazopanib<br>PD-173074<br>regorafenib<br>semaxanib<br>sorafenib<br>TG-100801<br>tivozanib<br>toceranib<br>vandetanib<br>vatalanib<br>ZM-306416	VEGF signling
FLT3	ENSG00000122025	AMG-925<br>BMS-690514<br>cediranib<br>ceritinib<br>crenolanib<br>dovitinib<br>ENMD-2076<br>gilteritinib<br>GTP-14564<br>HMN-214<br>KW-2449	VEGF signling

		lestaurtinib<br>linifanib<br>midostaurin<br>MK-2461<br>NMS-1286937<br>pacritinib<br>pexidartinib<br>ponatinib<br>quizartinib<br>rebastinib<br>RGB-286638<br>SGI-1776<br>sorafenib<br>tandutinib<br>TCS-359<br>TG-02<br>toceranib<br>tyrphostin-AG-1296<br>UNC2025<br>URMC-099	
FLT4	ENSG00000037280	axitinib<br>cediranib<br>dovitinib<br>foretinib<br>HMN-214<br>KRN-633<br>lenvatinib<br>linifanib<br>MK-2461<br>motesanib<br>nintedanib<br>pazopanib<br>PD-173074<br>regorafenib<br>SAR131675<br>sorafenib<br>tivozanib<br>vandetanib<br>vatalanib<br>XL-647<br>ZM-306416	VEGF signling
FRK	ENSG00000111816	bosutinib<br>dasatinib<br>regorafenib	Tyrosin kinase signaling
FYN	ENSG00000010810	bosutinib<br>dasatinib	Src Family Tyrosine Kinase
GLS	ENSG00000115419	CB-839<br>L-glutamic-acid	Phosphate-activated amidohydrolase
GSK3A	ENSG00000105723	AZD1080<br>CHIR-98014<br>CHIR-99021<br>GSK-3-inhibitor-IX	Wnt signaling pathway

		indirubin	
		SB-216763	
		SB-415286	
		AR-A014418	
		AT7867	
		AZD1080	
		AZD2858	
		bisindolylmaleimide-ix	
		BX-795	
		BX-912	
		CDK1-5-inhibitor	
		CHIR-98014	
		CHIR-99021	
		ellagic-acid	
		enzastaurin	
		GSK-3-inhibitor-IX	
		indirubin-3-monoxime	
		kenpaullone	
GSK3B	ENSG00000082701	LY2090314	Wnt signaling pathway
		LY294002	
		PD-98059	
		PF-573228	
		RGB-286638	
		SB-202190	
		SB-203580	
		SB-216763	
		SB-415286	
		TCS2002	
		tideglusib	
		TWS-119	
		U-0126	
		1-azakenpaullone	
		8-hydroxy-DPAT	
		ACY-1215	
		AR-42	
		belinostat	
		dacinostat	
		entinostat	
		givinostat	
		JNJ-26481585	
		mocetinostat	
		nexturastat-a	
HDAC1	ENSG00000116478	NSC-3852	Histone deacetylase
		panobinostat	
		parthenolide-(-)	
		PCI-24781	
		PCI-34051	
		phenylbutyrate	
		pivanex	
		pyroxamide	
		resminostat	
		RG2833	

		romidepsin<br>SB-939<br>scriptaid<br>sodium-butyrate<br>tacedinaline<br>trichostatin-a<br>valproic-acid<br>vorinostat<br>4SC-202	
HDAC2	ENSG00000196591	ACY-1215<br>aminophylline<br>belinostat<br>CUDC-907<br>dacinostat<br>entinostat<br>givinostat<br>JNJ-26481585<br>lovastatin<br>MC1568<br>mocetinostat<br>oxtriphylline<br>panobinostat<br>PCI-24781<br>pivanex<br>romidepsin<br>scriptaid<br>sodium-butyrate<br>trichostatin-a<br>valproic-acid<br>vorinostat	Histone deacetylase
HDAC3	ENSG00000171720	ACY-1215<br>belinostat<br>chidamide<br>dacinostat<br>entinostat<br>givinostat<br>JNJ-26481585<br>mocetinostat<br>panobinostat<br>PCI-24781<br>pivanex<br>resminostat<br>RGFP966<br>RG2833<br>romidepsin<br>SB-939<br>scriptaid<br>sodium-butyrate<br>trichostatin-a<br>vorinostat	Histone deacetylase
HDAC6	ENSG00000094631	ACY-1215<br>belinostat	Histone deacetylase

		bufexamac<br>dacinostat<br>droxinostat<br>givinostat<br>JNJ-26481585<br>nexturastat-a<br>panobinostat<br>PCI-24781<br>PCI-34051<br>resminostat<br>romidepsin<br>scriptaid<br>trichostatin-a<br>tubastatin-a<br>vorinostat	
HDAC8	ENSG00000147099	ACY-1215<br>belinostat<br>dacinostat<br>droxinostat<br>givinostat<br>JNJ-26481585<br>panobinostat<br>PCI-34051<br>pivanex<br>resminostat<br>romidepsin<br>scriptaid<br>sodium-butyrate<br>trichostatin-a<br>vorinostat	Histone deacetylase
HSP90AA1	ENSG00000080824	alvespimycin<br>AT13387<br>BIIB021<br>CCT018159<br>ganetespib<br>gedunin<br>geldanamycin<br>hexylene-glycol<br>nedocromil<br>NVP-AUY922<br>NVP-HSP990<br>PU-H71<br>retaspimycin<br>rhein<br>rifabutin<br>SNX-5422<br>tanespimycin<br>VER-49009	Heat Shock Protein 90 Alpha Family
IDH1	ENSG00000138413	AGI-5198<br>enasidenib<br>ivosidenib	Isocitrate dehydrogenase family
IDH2	ENSG00000182054	AGI-6780	Isocitrate dehydrogenase family

IGF1R	ENSG00000140443	AG-1024<br>AZD3463<br>AZD4547<br>BMS-536924<br>BMS-754807<br>ceritinib<br>GSK1838705A<br>GSK1904529A<br>linsitinib<br>NT157<br>NVP-ADW742<br>NVP-AEW541<br>podophyllotoxin<br>PQ-401<br>XL228	Receptor tyrosine kinase family
IL1B	ENSG00000125538	bergenin<br>diacerein<br>glucosamine<br>hypoestoxide<br>ibudilast<br>JTE-607<br>minocycline<br>tanshinone-iia	Interleukin-cytokine family
IL6	ENSG00000136244	ibudilast<br>JTE-607<br>trofinetide	Interleukin-cytokine family
INSR	ENSG00000171105	ceritinib<br>dovitinib<br>GSK1838705A<br>GSK1904529A<br>linsitinib<br>NVP-AEW541<br>NVP-TAE684	Receptor tyrosine kinase family
ITK	ENSG00000113263	pazopanib	Interleukin-cytokine family
JAK1	ENSG00000162434	AZD1480<br>baricitinib<br>curcumol<br>cyt387<br>filgotinib<br>itacitinib<br>NS-018<br>pacritinib<br>peficitinib<br>ruxolitinib<br>ruxolitinib-(S)<br>solcitinib<br>TG-101348<br>tofacitinib<br>WHI-P154<br>XL019<br>ZM-39923	Jak-STAT signaling pathway
JAK2	ENSG00000096968	AG-490	Jak-STAT signaling pathway

		AT-9283<br>atiprimod<br>AZD1480<br>AZ960<br>baricitinib<br>CEP-33779<br>CKD-712<br>curcumol<br>cyt387<br>filgotinib<br>LY2784544<br>NS-018<br>NVP-BSK805<br>pacritinib<br>peficitinib<br>RGB-286638<br>ruxolitinib<br>ruxolitinib-(S)<br>TG-02<br>TG-101209<br>TG-101348<br>thiram<br>tofacitinib<br>WHI-P154<br>WP1130<br>XL019	
JAK3	ENSG00000105639	AG-490<br>AT-9283<br>AZD1480<br>curcumol<br>cyt387<br>decernotinib<br>filgotinib<br>JAK3-inhibitor-V<br>LY2784544<br>NS-018<br>pacritinib<br>peficitinib<br>ruxolitinib<br>TG-101209<br>TG-101348<br>tofacitinib<br>WHI-P154<br>XL019<br>ZM-39923	Jak-STAT signaling pathway
KDM1A	ENSG00000004487	GSK2879552<br>OG-L002<br>ORY-1001<br>tranylcypromine	Histone demethylase
KDR	ENSG00000128052	ACTB-1003<br>AEE788	VEGF family

altiratinib
anlotinib
apatinib
axitinib
AZD4547
BMS-536924
BMS-690514
BMS-817378
brivanib
BX-795
BX-912
cabozantinib
cediranib
CYC116
dovitinib
ENMD-2076
foretinib
fruquintinib
golvatinib
HMN-214
KI-8751
KRN-633
lenvatinib
linifanib
lucitanib
LY2874455
MK-2461
motesanib
neratinib
nintedanib
NVP-BGJ398
orantinib
OSI-930
pazopanib
PD-153035
PD-173074
PLX-4720
ponatinib
PP-121
regorafenib
semaxanib
SKLB1002
sorafenib
SU-4312
SU014813
telatinib
TG-100572
TG-100801
tie2-kinase-inhibitor
tivozanib
toceranib
tyrphostin-AG-879
vandetanib

		vatalanib<br>XL-647<br>ZM-306416<br>ZM-323881	
KIT	ENSG00000157404	amuvatinib<br>apatinib<br>cediranib<br>CP-673451<br>crenolanib<br>dasatinib<br>DCC-2618<br>dovitinib<br>GTP-14564<br>HMN-214<br>imatinib<br>linifanib<br>lucitanib<br>masitinib<br>motesanib<br>nilotinib<br>OSI-930<br>pazopanib<br>pexidartinib<br>ponatinib<br>quizartinib<br>regorafenib<br>semaxanib<br>sorafenib<br>tandutinib<br>tivozanib	RAS-ERK signaling pathway
LAP3	ENSG00000002549	tosedostat	Leucine Aminopeptidase
LCK	ENSG00000182866	bisindolylmaleimide-ix<br>BMS-536924<br>CGP-57380<br>dasatinib<br>GW-788388<br>KN-62<br>LY294002<br>PD-98059<br>ponatinib<br>PP-2<br>saracatinib<br>SB-202190<br>SB-203580<br>SRC-kinase-inhibitor-I<br>tozasertib<br>U-0126<br>ZM-336372<br>8-hydroxy-DPAT	Src Family Tyrosine Kinase
LDHA	ENSG00000134333	coenzyme-i<br>nicotinamide	Lactate Dehydrogenase

LYN	ENSG00000254087	bafetinib<br>bosutinib<br>dasatinib<br>ponatinib<br>PP-2<br>rebastinib<br>tolimidone	Src Family Tyrosine Kinase
MAP2	ENSG00000078018	docetaxel<br>estramustine<br>estramustine-phosphate<br>paclitaxel	Microtubule Associated Protein
MAP2K1	ENSG00000169032	arctigenin<br>AS-703026<br>BI-847325<br>bosutinib<br>MEK162<br>nobiletin<br>PD-0325901<br>PD-184352<br>PD-198306<br>PD-318088<br>PD-98059<br>refametinib<br>reversine<br>Ro-4987655<br>Ro-5126766<br>selumetinib<br>TAK-733<br>trametinib<br>U-0126	RAS-ERK signaling pathway
MAP2K2	ENSG00000126934	AS-703026<br>BI-847325<br>bosutinib<br>MEK1-2-inhibitor<br>MEK162<br>PD-198306<br>refametinib<br>Ro-5126766<br>trametinib<br>U-0126	RAS-ERK signaling pathway
MAP3K8	ENSG00000107968	cot-inhibitor-1<br>cot-inhibitor-2	RAS-ERK signaling pathway
MAP4	ENSG00000047849	docetaxel<br>paclitaxel	Microtubule Associated Protein
MAPK1	ENSG00000100030	arsenic-trioxide<br>bisindolylmaleimide-ix<br>BMS-536924<br>CHIR-99021<br>FR-180204<br>GDC-0994<br>isoprenaline	RAS-ERK signaling pathway

		KN-62<br>LY294002<br>olomoucine<br>PD-98059<br>purvalanol-b<br>ravoxertinib<br>SB-202190<br>SB-203580<br>TIC10<br>U-0126<br>VX-11e	
MAPK11	ENSG00000185386	AMG-548<br>bisindolylmaleimide-ix<br>doramapimod<br>EO-1428<br>KN-62<br>LY294002<br>PD-98059<br>PH-797804<br>regorafenib<br>SB-202190<br>SB-203580<br>SB-239063<br>talmapimod<br>U-0126<br>VX-702<br>VX-745	RAS-ERK signaling pathway
MAPK12	ENSG00000188130	AMG-548<br>bisindolylmaleimide-ix<br>doramapimod<br>KN-62<br>LY294002<br>PD-98059<br>SB-202190<br>SB-203580<br>U-0126<br>VX-702<br>VX-745	RAS-ERK signaling pathway
MAPK13	ENSG00000156711	AMG-548<br>doramapimod	RAS-ERK signaling pathway
MAPK14	ENSG00000112062	AMG-548<br>bisindolylmaleimide-ix<br>CMPD-1<br>doramapimod<br>EO-1428<br>GW-788388<br>JX-401<br>KN-62<br>losmapimod<br>LY2228820<br>LY294002	RAS-ERK signaling pathway

		PD-98059<br>pexmetinib<br>PH-797804<br>QX-314<br>RWJ-67657<br>SB-202190<br>SB-203580<br>SB-239063<br>SB-242235<br>SD-169<br>semapimod<br>skepinone-l<br>SKF-86002<br>SX-011<br>TA-01<br>TAK-715<br>talmapimod<br>tie2-kinase-inhibitor<br>tyrphostin-AG-1478<br>U-0126<br>VX-702<br>VX-745<br>ZM-336372<br>8-hydroxy-DPAT	
MAPK3	ENSG00000102882	arsenic-trioxide<br>CID-5458317<br>FR-180204<br>GDC-0994<br>purvalanol-b<br>ravoxertinib<br>sulindac	RAS-ERK signaling pathway
MAPK7	ENSG00000166484	BIX-02189<br>ERK5-IN-1<br>XMD17-109<br>XMD8-92	RAS-ERK signaling pathway
MAPK8	ENSG00000107643	antagonist-g<br>BI-78D3<br>bisindolylmaleimide-ix<br>CC-401<br>KN-62<br>LY294002<br>PD-98059<br>PGL5001<br>pyrazolanthrone<br>SB-202190<br>SB-203580<br>SR-3306<br>SU3327<br>U-0126	RAS-ERK signaling pathway
MAPK9	ENSG00000050748	PGL5001<br>pyrazolanthrone	RAS-ERK signaling pathway

		RGB-286638<br>SB-203580<br>SR-3306	
MAPT	ENSG00000186868	docetaxel<br>leucomethylene-blue<br>paclitaxel	Microtubule Associated Protein
MCL1	ENSG00000143384	bithionol<br>morin<br>rosmarinic-acid<br>TW-37	BCL2 Family Member
MDM2	ENSG00000135679	AMG-232<br>CGM097<br>idasanutlin<br>JNJ-26481585<br>nutlin-3<br>RG7112<br>RITA<br>SAR405838<br>serdemetan	TP53-MDM2 pathway
MET	ENSG00000105976	alectinib<br>altiratinib<br>AMG-208<br>AMG-337<br>AMG458<br>amuvatinib<br>BMS-536924<br>BMS-777607<br>BMS-817378<br>cabozantinib<br>crizotinib<br>EMD-1214063<br>foretinib<br>golvatinib<br>INC-280<br>JNJ-38877605<br>LY2801653<br>MGCD-265<br>MK-2461<br>MK-8033<br>NVP-BVU972<br>PF-04217903<br>PHA-665752<br>savolitinib<br>SGX523<br>SU-11274<br>tivantinib	RAS-ERK signaling pathway
MKNK1	ENSG00000079277	CGP-57380	
MTOR	ENSG00000198793	AZD2014<br>AZD8055<br>AZ20<br>beta-hydroxy-beta-	HGF-MET-PI3K signaling pathway

		methylbutyrate<br>BGT226<br>CC-115<br>CC-223<br>chrysophanic-acid<br>CH5132799<br>compound-401<br>deforolimus<br>everolimus<br>GDC-0980<br>GSK2126458<br>ku-0063794<br>LY294002<br>LY3023414<br>MLN0128<br>NVP-BEZ235<br>OSI-027<br>palomid-529<br>PF-04691502<br>PF-05212384<br>PI-103<br>pimecrolimus<br>PKI-179<br>PP-121<br>PP242<br>SAR-245409<br>SB-2343<br>sirolimus<br>temsirolimus<br>torin-1<br>torin-2<br>VE-822<br>voxtalisib<br>WAY-600<br>WYE-125132<br>WYE-354<br>XL388	
MYC	ENSG00000136997	TWS-119	MYC family
NAMPT	ENSG00000105835	FK-866<br>GMX1778<br>STF-118804	Nicotinamide Phosphoribosyltransferase
NFKB1	ENSG00000109320	andrographolide<br>arglabin<br>aspirin<br>pranlukast<br>thalidomide<br>triflusal	NFKB pathway
NR2C2	ENSG00000177463	retinol<br>tretinoin	Hormone receptor family
NTRK1	ENSG00000198400	amitriptyline<br>danusertib	Tyrosine kinase receptor family

		entrectinib<br>GW-441756<br>GW-5074<br>imatinib<br>lestaurtinib<br>LOXO-101<br>MK-2461<br>PHA-848125<br>regorafenib<br>tyrphostin-AG-879	
NUDT1	ENSG00000106268	crizotinib-(S)<br>TH588	Hydrolase family
P4HB	ENSG00000185624	ribostamycin-sulfate	Hydroxylase famliy
PAK1	ENSG00000149269	FRAX486<br>IPA-3<br>RKI-1447	PAK family - P21 activated kinase
PAK4	ENSG00000130669	FRAX486<br>GSK690693<br>PF-03758309	PAK family - P21 activated kinase
PARP1	ENSG00000143799	AG-14361<br>BYK-204165<br>DR-2313<br>E7449<br>iniparib<br>inosine<br>nicotinamide<br>niraparib<br>NU-1025<br>olaparib<br>PJ-34<br>rucaparib<br>S-111<br>veliparib<br>3-amino-benzamide<br>4-HQN	TP53-MDM2 pathway
PARP2	ENSG00000129484	AC-55541<br>E7449<br>olaparib<br>rucaparib<br>talazoparib<br>UPF-1069<br>veliparib	TP53-MDM2 pathway
PARP3	ENSG00000041880	DR-2313<br>ME0328<br>PJ-34	TP53-MDM2 pathway
PDGFRA	ENSG00000134853	BMS-536924<br>cediranib<br>CP-673451<br>crenolanib<br>DCC-2618<br>dovitinib<br>ENMD-2076	PDGF-PDGFR-RAS-ERK signaling pathway

		HMN-214<br>imatinib<br>lucitanib<br>masitinib<br>nintedanib<br>orantinib<br>pazopanib<br>PD-173074<br>ponatinib<br>PP-121<br>quizartinib<br>regorafenib<br>semaxanib<br>TAK-593<br>tivozanib<br>toceranib<br>trapidil	
PDGFRB	ENSG00000113721	anlotinib<br>BMS-536924<br>cediranib<br>CP-673451<br>crenolanib<br>dasatinib<br>dovitinib<br>flumatinib<br>GTP-14564<br>HMN-214<br>imatinib<br>linifanib<br>lucitanib<br>masitinib<br>MK-2461<br>nintedanib<br>ON123300<br>orantinib<br>pazopanib<br>PD-173074<br>quizartinib<br>regorafenib<br>semaxanib<br>sorafenib<br>SU-4312<br>tandutinib<br>tivozanib<br>toceranib<br>vatalanib	PDGF-PDGFR-RAS-ERK signaling pathway
PDK1	ENSG00000152256	AZD7545<br>BX-795<br>BX-912<br>dichloroacetate	PI3K signaling pathway
PGD	ENSG00000142657	dacarbazine	Phosphogluconate Dehydrogenase

		ketotifen<br>pentetic-acid	
PIK3CA	ENSG00000121879	alpelisib<br>AS-604850<br>AZD6482<br>AZD8835<br>A66<br>BGT226<br>buparlisib<br>caffeine<br>CH5132799<br>copanlisib<br>GDC-0349<br>GDC-0941<br>GDC-0980<br>GSK2126458<br>idelalisib<br>IPI-145<br>LY294002<br>MLN0128<br>MLN1117<br>NVP-BEZ235<br>PF-04691502<br>PF-05212384<br>PI-103<br>PIK-293<br>PIK-75<br>PP-121<br>SB-2343<br>taselisib<br>TG100-115<br>voxtalisib<br>wortmannin<br>XL-147	HGF-MET-PI3K signaling pathway
PIK3CB	ENSG00000051382	acalisib<br>alpelisib<br>AZD6482<br>AZD8186<br>BGT226<br>caffeine<br>CH5132799<br>copanlisib<br>GDC-0941<br>GDC-0980<br>GSK2126458<br>GSK2636771<br>idelalisib<br>IPI-145<br>LY294002<br>PI-103<br>PIK-293<br>PIK-294	HGF-MET-PI3K signaling pathway

		PIK-75<br>PP-121<br>SB-2343<br>TGX-221<br>TG100-115<br>ZSTK-474	
PIK3CD	ENSG00000171608	acalisib<br>alpelisib<br>AMG-319<br>AMG319<br>AZD6482<br>AZD8186<br>AZD8835<br>caffeine<br>CH5132799<br>copanlisib<br>GDC-0941<br>GDC-0980<br>GSK2126458<br>idelalisib<br>IPI-145<br>LY294002<br>MLN0128<br>NVP-BEZ235<br>PI-103<br>PIK-293<br>PIK-294<br>PIK-75<br>PP-121<br>SB-2343<br>TGX-221<br>TG100-115<br>wortmannin<br>XL-147<br>ZSTK-474	HGF-MET-PI3K signaling pathway
PIK3CG	ENSG00000105851	alpelisib<br>AS-252424<br>AS-604850<br>AZD6482<br>BGT226<br>buparlisib<br>CAY10505<br>CH5132799<br>copanlisib<br>CZC24832<br>GDC-0941<br>GDC-0980<br>GSK1059615<br>GSK2126458<br>idelalisib<br>IPI-145<br>LY294002	HGF-MET-PI3K signaling pathway

		MLN0128<br>myricetin<br>NVP-BEZ235<br>PI-103<br>PIK-293<br>PIK-294<br>PIK-75<br>PIK-93<br>PP-121<br>quercetin<br>SAR-245409<br>SB-2343<br>TG100-115<br>VE-822<br>wortmannin<br>XL-147<br>ZSTK-474	
PIM1	ENSG00000137193	adenosine-phosphate<br>ammonium-perfluorocaprylate<br>AZD1208<br>BMS-863233<br>dexfosfoserine<br>GF109203X<br>PIM-1-Inhibitor-2<br>quercetin<br>SGI-1776<br>SMI-4a<br>10-DEBC	Serine/Threonine Kinase family
PIM2	ENSG00000102096	AZD1208<br>SGI-1776	Serine/Threonine Kinase family
PIM3	ENSG00000198355	AZD1208<br>SGI-1776	Serine/Threonine Kinase family
PLK1	ENSG00000166851	BI-2536<br>GSK461364<br>GW-843682X<br>LY294002<br>NMS-1286937<br>rigosertib<br>TAK-960<br>volasertib<br>wortmannin	Serine/threonine kinase family
PORCN	ENSG00000102312	ETC-159<br>IWP-L6<br>LGK-974<br>wnt-c59	WNT signaling pathway
PPM1D	ENSG00000170836	GSK2830371	Serine/threonine kinase family
PRKAA1	ENSG00000132356	adenosine-phosphate<br>adenosine-triphosphate<br>aspirin<br>phenformin	Serine/threonine kinase family
PRKCA	ENSG00000154229	bisindolylmaleimide-ix	Serine/threonine kinase family

		C-1<br>ellagic-acid<br>enzastaurin<br>go-6983<br>ingenol-mebutate<br>KN-62<br>LY294002<br>myricitrin<br>PD-98059<br>SB-202190<br>SB-203580<br>SC-10<br>SC-9<br>sotrastaurin<br>tamoxifen<br>U-0126<br>vitamin-E<br>8-hydroxy-DPAT	
PRKCB	ENSG00000166501	CGP-53353<br>ellagic-acid<br>enzastaurin<br>go-6983<br>ingenol-mebutate<br>sotrastaurin<br>tamoxifen<br>vitamin-E<br>8-hydroxy-DPAT	Serine/threonine kinase family
PRKCD	ENSG00000163932	afimoxifene<br>enzastaurin<br>go-6983<br>ingenol-mebutate<br>sotrastaurin<br>tamoxifen<br>8-hydroxy-DPAT	Serine/threonine kinase family
PRKCE	ENSG00000171132	afimoxifene<br>BMY-45618<br>ingenol-mebutate<br>sotrastaurin<br>tamoxifen<br>Y-27632<br>8-hydroxy-DPAT	Serine/threonine kinase family
PRKCG	ENSG00000126583	enzastaurin<br>go-6983<br>ingenol-mebutate<br>midostaurin<br>tamoxifen<br>8-hydroxy-DPAT	Serine/threonine kinase family
PRKCH	ENSG00000027075	sotrastaurin	Serine/threonine kinase family
PRKCI	ENSG00000163558	aurothioglucose<br>GF109203X<br>tamoxifen	Serine/threonine kinase family

PRKCQ	ENSG00000065675	afimoxifene<br>dexfosfoserine<br>GSK690693<br>sotrastaurin<br>tamoxifen	Serine/threonine kinase family
PRKCZ	ENSG00000067606	afimoxifene<br>arachidonic-acid<br>GF109203X<br>go-6983<br>tamoxifen	Serine/threonine kinase family
PRKDC	ENSG00000253729	caffeine<br>compound-401<br>KU-55933<br>LY294002<br>NU-7026<br>NU-7441<br>PI-103<br>PIK-75<br>PP-121<br>wortmannin	Serine/threonine kinase family
PSMB1	ENSG00000008018	bortezomib<br>carfilzomib<br>MG-132	Multicatalytic proteinase complex
PSMD1	ENSG00000173692	bortezomib	Multicatalytic proteinase complex
PTGS2	ENSG00000073756	aceclofenac<br>acemetacin<br>alpha-linolenic-acid<br>amfenac<br>aminosalicylate<br>ampiroxicam<br>amtolmetin-guacil<br>arundic-acid<br>asaraldehyde<br>aspirin<br>azapropazone<br>balsalazide<br>bendazac<br>bromfenac<br>carprofen<br>celecoxib<br>cicloprofen<br>curcumin<br>deracoxib<br>diclofenac<br>diflunisal<br>dihomo-gamma-linolenic-acid<br>doconexent<br>DUP-697<br>epirizole<br>etodolac<br>etofenamate	Prostaglandin-endoperoxide synthase family

etoricoxib
felbinac-ethyl
fenbufen
fenoprofen
fentiazac
firocoxib
FK-3311
floctafenine
flufenamic-acid
flurbiprofen-(+/-)
flurbiprofen-(S)-(+)
fosfosal
gamma-linolenic-acid
ginsenoside-c-k
honokiol
ibuprofen
ibuprofen-(s)
ibuprofen-lysine
ibuprofen-piconol
icosapent
iguratimod
indobufen
indomethacin
indoprofen
isoxicam
ketoprofen
ketorolac
lenalidomide
licofelone
lornoxicam
loxoprofen
lumiracoxib
meclofenamic-acid
mefenamic-acid
meloxicam
mesalazine
metoxibutropate
nabumetone
naproxen
nepafenac
niflumic-acid
nimesulide
NO-ASA
oxaprozin
oxyphenbutazone
paracetamol
phenazone
phenylbutazone
piroxicam
pomalidomide
proglumetacin

		propacetamol<br>proquazone<br>pterostilbene<br>resveratrol<br>rofecoxib<br>rutaecarpine<br>RWJ-67657<br>salicylic-acid<br>sasapyrine<br>SB-239063<br>sodium-salicylate<br>sulfasalazine<br>sulindac<br>suprofen<br>tenoxicam<br>thalidomide<br>thioctic-acid<br>tiaprofenic-acid<br>tolmetin<br>tropesin<br>ufenamate<br>valdecoxib	
PTK2	ENSG00000169398	defactinib<br>ENMD-2076<br>NVP-TAE226<br>PF-562271	Serine/threonine kinase family
PTPN11	ENSG00000179295	BVT-948	Protein tyrosine phosphatase (PTP) family
PTPN6	ENSG00000111679	sodium-stibogluconate	Protein tyrosine phosphatase (PTP) family
RAC1	ENSG00000136238	dextromethorphan<br>EHop-016	RAS superfamily
RAF1	ENSG00000132155	AZ-628<br>CEP-32496<br>dabrafenib<br>GW-5074<br>LY3009120<br>PD-98059<br>regorafenib<br>Ro-5126766<br>SB-203580<br>sorafenib<br>U-0126<br>vemurafenib<br>ZM-336372	EGFR-RAS-ERK signaling pathway
RARA	ENSG00000131759	AC-55649<br>acitretin<br>adapalene<br>AGN-195183<br>AM-580<br>CH55<br>EC-23<br>etretinate	Retinoic acid signaling pathway

		fenretinide<br>isotretinoin<br>tamibarotene<br>tazarotene<br>tretinoin<br>TTNPB	
RARB	ENSG00000077092	AC-55649<br>acitretin<br>adapalene<br>adarotene<br>CH55<br>EC-23<br>etretinate<br>isotretinoin<br>LE-135<br>tamibarotene<br>tazarotene<br>tretinoin<br>TTNPB	Retinoic acid signaling pathway
RARG	ENSG00000172819	acitretin<br>adapalene<br>adarotene<br>CD-1530<br>CD-437<br>EC-23<br>etretinate<br>isotretinoin<br>palovarotene<br>tazarotene<br>tretinoin<br>TTNPB	Retinoic acid signaling pathway
RET	ENSG00000165731	apatinib<br>danusertib<br>fostamatinib<br>HMN-214<br>imatinib<br>linifanib<br>lucitanib<br>ponatinib<br>PP-1<br>quizartinib<br>regorafenib<br>semaxanib<br>sorafenib<br>vandetanib	RAS-ERK signaling pathway
ROCK1	ENSG00000067900	AT13148<br>bisindolylmaleimide-ix<br>fasudil<br>GSK429286A<br>hydroxyfasudil<br>KN-62<br>LX7101	RhoGEF pathway

		LY294002<br>PD-98059<br>ripasudil<br>RKI-1447<br>SAR407899<br>SB-202190<br>SB-203580<br>U-0126<br>Y-27632<br>Y-39983	
ROCK2	ENSG00000134318	AT13148<br>fasudil<br>KD025<br>LX7101<br>ripasudil<br>RKI-1447<br>Y-27632<br>Y-39983	RhoGEF pathway
RPS6KB1	ENSG00000108443	bisindolylmaleimide-ix<br>KN-62<br>LY2584702<br>LY294002<br>PD-98059<br>PF-4708671<br>PHA-767491<br>SB-202190<br>SB-203580<br>SB-415286<br>U-0126	PI3K-AKT-MTOR pathway
RRM1	ENSG00000167325	caracemide<br>cladribine<br>clofarabine<br>didox<br>enocitabine<br>fludarabine<br>fludarabine-phosphate<br>gemcitabine<br>gemcitabine-elaidate<br>hydroxyurea<br>imexon<br>triapine	RAS-ERK signaling pathway
RXRA	ENSG00000186350	acitretin<br>adapalene<br>AGN-194310<br>bexarotene<br>BMS-649<br>etodolac<br>etretinate<br>GW-9662<br>peretinoin<br>retinol	Retinoic acid signaling pathway

RXRB	ENSG00000204231	acitretin<br>adapalene<br>AGN-194310<br>bexarotene<br>etretinate<br>retinol<br>T-0901317<br>tazarotene<br>tretinoin	Retinoic acid signaling pathway
SGK3	ENSG00000104205	AT13148	PI3K-AKT-MTOR pathway
SMO	ENSG00000128602	BMS-833923<br>CUR-61414<br>fluocinonide<br>glasdegib<br>PF-5274857<br>purmorphamine<br>SANT-1<br>SANT-2<br>sonidegib<br>taladegib<br>vismodegib	Hedgehog signaling pathway
SRC	ENSG00000197122	bosutinib<br>citric-acid<br>dasatinib<br>ENMD-2076<br>KX2-391<br>PD-168393<br>ponatinib<br>PP-121<br>PP-2<br>purvalanol-a<br>rebastinib<br>saracatinib<br>SB-203580<br>TG-100572<br>TG-100801<br>tolimidone<br>vandetanib<br>XL228<br>1-naphthyl-PP1	SRC pathway
STAT3	ENSG00000168610	acitretin<br>atiprimod<br>bardoxolone-methyl<br>cryptotanshinone<br>meisoindigo<br>napabucasin<br>niclosamide<br>ochromycinone<br>STAT3-inhibitor-VI<br>WP1066	Jak-STAT signaling pathway
SYK	ENSG00000165025	BAY-61-3606<br>ellagic-acid	TLR signaling

		ER-27319<br>fostamatinib<br>GDC-0980<br>GS-9973<br>PRT062070<br>PRT062607<br>R112	
TEK	ENSG00000120156	ACTB-1003<br>altiratinib<br>linifanib<br>pexmetinib<br>ponatinib<br>regorafenib<br>vandetanib	Tie2 family
TNF	ENSG00000232810	amrinone<br>baicalein<br>bergenin<br>chloroquine<br>clenbuterol<br>CPI-1189<br>epinephrine<br>ethyl-pyruvate<br>glucosamine<br>glycitein<br>hypoestoxide<br>JTE-607<br>lenalidomide<br>ligustilide<br>pentoxifylline<br>pirfenidone<br>pomalidomide<br>pranlukast<br>SB-203580<br>tanshinone-iia<br>thalidomide<br>trofinetide	TNF signaling pathway
TNFSF11	ENSG00000120659	lenalidomide	TNF signaling pathway
TNFSF13B	ENSG00000102524	citric-acid	TNF signaling pathway
TOP1	ENSG00000198900	aclarubicin<br>beta-lapachone<br>camptothecin<br>exatecan-mesylate<br>genz-644282<br>irinotecan<br>luteolin<br>nemorubicin<br>pyrazoloacridine<br>rubitecan<br>SN-38<br>sodium-stibogluconate<br>TAS-103	Topoisomerase family

		topotecan<br>10-hydroxycamptothecin<br>9-aminocamptothecin	
TOP2A	ENSG00000131747	aclarubicin<br>aldoxorubicin<br>amonafide<br>amrubicin<br>amsacrine<br>ciprofloxacin<br>daunorubicin<br>dexrazoxane<br>doxorubicin<br>enoxacin<br>epirubicin<br>etoposide<br>etoposide-phosphate<br>garenoxacin<br>genistein<br>idarubicin<br>levofloxacin<br>lomefloxacin<br>merbarone<br>mitoxantrone<br>moxifloxacin<br>nemorubicin<br>norfloxacin<br>ofloxacin<br>pefloxacin<br>pirarubicin<br>pixantrone<br>podophyllotoxin<br>pyrazoloacridine<br>sparfloxacin<br>teniposide<br>trovafloxacin<br>valrubicin<br>voreloxin	Topoisomerase family
TOP2B	ENSG00000077097	amonafide<br>amrubicin<br>daunorubicin<br>dexrazoxane<br>etoposide<br>teniposide	Topoisomerase family
TRPV1	ENSG00000196689	allicin<br>alpha-linolenic-acid<br>AM-404<br>AMG-517<br>AMG-9810<br>anandamide<br>aspartame<br>camphor-(+)	TRP family of ion channels

		capsaicin	
		capsazepine	
		evodiamine	
		icosapent	
		JNJ-17203212	
		MK-2295	
		nonivamide	
		olvanil	
		paracetamol	
		piperine	
		SB-366791	
		SB-705498	
		2-APB	
		6-iodo-nordihydrocapsaicin	
TUBB	ENSG00000196230	ABT-751	Microtubule Associated Protein
		albendazole	
		cabazitaxel	
		carbendazim	
		cevipabulin	
		colchicine	
		CYT-997	
		D-64131	
		docetaxel	
		dolastatin-10	
		epothilone-b	
		epothilone-d	
		fenbendazole	
		flubendazole	
		ixabepilone	
		mebendazole	
		oxibendazole	
		paclitaxel	
		parbendazole	
		podophyllotoxin	
		verubulin	
		vinblastine	
		vincristine	
		vindesine	
		vinorelbine	
		2-methoxyestradiol	
TXN	ENSG00000136810	C11-Acetate	Redox signaling
		PX-12	
TYMS	ENSG00000176890	capecitabine	Thymidylate synthase
		carmofur	
		doxifluridine	
		enocitabine	
		floxuridine	
		ftorafur	
		gemcitabine	
		gemcitabine-elaidate	
		leucovorin	
		nolatrexed	

		pemetrexed<br>pralatrexate<br>raltitrexed<br>trifluridine<br>trimethoprim<br>WR99210<br>5-fluorouracil<br>5-FP	
TYRO3	ENSG00000092445	BMS-777607<br>LDC1267	Protein Tyrosine Kinase family
VEGFA	ENSG00000112715	carvedilol<br>gliclazide<br>minocycline<br>pidolic-acid<br>trometamol<br>vandetanib	VEGF family
WEE1	ENSG00000166483	MK-1775<br>PD-407824	Serine/Threonine Kinase family
XIAP	ENSG00000101966	AZD5582<br>birinapant<br>cisplatin<br>CUDC-427<br>embelin<br>GDC-0152<br>LCL-161<br>SM-164	BCL2 signaling
XPO1	ENSG00000082898	elactocin<br>KPT-185<br>KPT-276<br>selinexor	Cell cycle regulator

Supplementary Table S1B. shRNAs targeting each candidate gene are reported. For each cell line sequenced, LOG_2 ratio of each replica calculated; call for depletion in all tree replicates; gene depletion call.

Cell line	shRNAs	LOG2R(1)	LOG2R(2)	LOG2R(3)	Triplicate shRNA Hit
CAR1	BIRC5_1908	-5,85	-7,39	-4,04	0
	BIRC5_1909	-6,21	-6,91	-7,64	1
	BIRC5_1910	-8,56	-7,15	-6,91	1
	BIRC5_1911	1,15	-1,89	-0,95	0
	BIRC5_1912	-0,96	-7,14	-5,28	0
	BIRC5_1913	-0,68	-2,55	-2,59	0
	BIRC5_599	-8,87	-7,51	-8,20	1
	BIRC5_600	-7,96	-7,55	-7,42	1
	BIRC5_601	-7,39	-7,82	-4,23	0
	BIRC5_602	-10,89	1,81	-9,36	0
CAR1	EGFR_1660	-3,43	-2,36	-11,33	0
	EGFR_1701	-9,64	-7,98	-9,40	1
	EGFR_1753	-10,06	-8,28	-9,61	1
	EGFR_716	3,74	-7,11	-3,36	0
	EGFR_717	-10,14	-7,83	-7,25	1
	EGFR_718	-1,22	-9,98	-4,53	0
	EGFR_719	-0,22	-8,64	-10,63	0
	EGFR_720	-10,92	-10,02	-7,31	1
	EGFR_721	-3,38	-5,43	-5,25	0
	EGFR_722	-5,05	-4,10	-4,77	0
CAR1	LAP3_2092	-4,25	-1,21	-5,51	0
	LAP3_2093	-2,38	-2,40	-1,41	0
	LAP3_2094	-11,38	-9,06	-9,13	1
	LAP3_2095	-4,07	-3,46	-12,70	0
	LAP3_2096	-10,42	-8,18	-8,59	1
	LAP3_947	-5,02	-3,89	-2,28	0
	LAP3_948	-6,16	-9,58	-10,06	1
	LAP3_949	-3,21	-3,77	-1,94	0
	LAP3_950	-11,14	-6,28	-7,04	1
	LAP3_951	-4,64	-11,95	-2,35	0
WIDR	BRAF_1610	1,08	-1,00	-0,63	0
	BRAF_1795	-2,17	-1,26	-1,27	1
	BRAF_613	-1,52	-1,35	-3,85	1
	BRAF_614	-0,37	-3,56	-0,33	0
	BRAF_615	-5,58	-1,01	1,24	0
	BRAF_616	-1,93	-1,96	-1,75	1
	BRAF_617	-2,46	-0,72	-0,83	0
	BRAF_618	-6,94	-3,61	0,35	0
	BRAF_619	-1,34	-1,58	-1,53	1
	BRAF_620	-1,84	-7,33	-3,94	1
WIDR	GLS_2030	0,64	0,00	0,21	0
	GLS_2031	-0,52	0,13	-0,61	0
	GLS_2032	0,40	0,61	-0,91	0
	GLS_2033	-1,08	-1,54	-1,12	1
	GLS_2034	-0,68	-0,55	-1,01	0
	GLS_807	-2,54	-3,38	-2,13	1
	GLS_808	1,06	1,06	0,64	0

	GLS_809	-3,07	-3,22	-3,34	1
	GLS_810	-5,07	-1,95	-2,15	1
	GLS_811	-0,33	-1,25	-0,89	0
WIDR	HDAC8_1830	-3,21	-4,17	-1,24	1
	HDAC8_2042	1,55	1,40	1,52	0
	HDAC8_2043	-0,37	-2,47	-1,62	0
	HDAC8_2044	-2,99	-1,64	-2,51	1
	HDAC8_2045	-0,84	-0,18	-0,45	0
	HDAC8_856	1,11	0,82	0,28	0
	HDAC8_857	-2,04	-3,05	-1,12	1
	HDAC8_858	1,07	1,58	0,89	0
	HDAC8_859	-0,97	-0,87	-0,70	0
	HDAC8_860	-2,92	-1,50	-3,42	1
WIDR	MAPK1_1004	-1,02	-0,45	-1,17	0
	MAPK1_1005	-1,09	-1,07	-0,85	1
	MAPK1_1006	-1,53	-2,61	-1,32	1
	MAPK1_1007	-0,80	-0,46	-0,40	0
	MAPK1_1008	-2,47	-1,45	-1,84	1
	MAPK1_1009	-1,88	-2,19	0,18	0
	MAPK1_1010	-1,54	0,44	-0,01	0
	MAPK1_1717	-0,21	-0,13	0,02	0
	MAPK1_2122	-0,03	-4,17	-3,95	0
	MAPK1_2123	-1,70	-1,73	-2,51	1
WIDR	PRKAA1_1238	-1,10	-2,56	-2,27	1
	PRKAA1_1239	-1,43	1,40	-1,58	0
	PRKAA1_1240	-2,00	-1,11	-1,04	1
	PRKAA1_1241	-1,37	-1,70	-1,46	1
	PRKAA1_1539	0,90	0,80	0,79	0
	PRKAA1_1540	0,20	-1,47	-1,33	0
	PRKAA1_1541	-1,72	-2,43	-1,33	1
	PRKAA1_1789	2,69	2,75	2,51	0
	PRKAA1_1790	-0,57	-1,43	-2,07	0
	PRKAA1_1791	1,63	1,12	1,14	0
WIDR	PRKCI_1267	-1,39	-0,53	-1,91	0
	PRKCI_1268	-0,37	0,23	-0,04	0
	PRKCI_1269	-2,58	-1,78	-2,55	1
	PRKCI_1270	-1,46	-1,81	-1,87	1
	PRKCI_1271	-1,15	-1,69	-0,89	1
	PRKCI_1272	-1,03	-1,50	0,99	0
	PRKCI_1604	3,37	4,24	4,31	0
	PRKCI_1605	-0,71	1,25	0,42	0
	PRKCI_1787	1,53	0,83	1,12	0
	PRKCI_2255	-3,26	-1,26	-2,01	1
LIM2099	CDK1_1944	2,92	2,57	0,93	0
	CDK1_1945	0,96	0,30	0,14	0
	CDK1_1946	-5,39	-1,58	-3,55	1
	CDK1_1947	-3,06	0,55	-0,84	1
	CDK1_1948	-7,33	6,56	-1,14	0
	CDK1_1949	0,20	0,62	-0,21	0
	CDK1_1950	-0,02	2,77	1,88	0
	CDK1_641	-2,44	-2,94	-1,81	1

	CDK1_642	-4,85	-5,44	-4,28	1
	CDK1_643	0,57	0,55	0,21	0
LIM2099	FYN_1575	0,05	5,10	3,93	0
	FYN_1576	-2,57	-1,69	-5,52	1
	FYN_1833	0,82	-0,60	-0,34	0
	FYN_1834	2,43	2,00	2,29	0
	FYN_801	1,28	2,27	1,21	0
	FYN_802	-2,56	0,64	-3,90	1
	FYN_803	-1,29	2,82	1,94	0
	FYN_804	-0,79	5,71	0,82	0
	FYN_805	-1,54	-1,40	-1,40	1
	FYN_806	-5,47	-1,46	-1,66	1
SW403	CDK1_1944	0,01	-0,11	0,53	0
	CDK1_1945	1,14	1,38	0,84	0
	CDK1_1946	-3,15	-0,13	-2,52	0
	CDK1_1947	-0,65	0,81	-1,54	0
	CDK1_1948	-9,31	-8,88	-8,54	1
	CDK1_1949	-5,06	-3,30	0,84	0
	CDK1_1950	-1,50	-3,18	-8,13	1
	CDK1_641	-2,91	-2,39	-1,08	1
	CDK1_642	-1,95	3,30	-8,36	0
	CDK1_643	-1,21	-3,64	-2,06	1
SW403	CDK4_1508	3,24	-0,19	-2,38	0
	CDK4_1734	4,76	-9,70	-0,59	0
	CDK4_652	-5,72	-2,68	-1,88	1
	CDK4_653	-0,90	-5,22	-3,27	1
	CDK4_654	6,32	-0,71	-6,10	0
	CDK4_655	-9,62	-9,52	-1,49	1
	CDK4_656	-0,20	-0,06	0,99	0
	CDK4_657	-0,57	-2,75	-1,23	0
	CDK4_658	0,88	1,40	-7,44	0
	CDK4_659	-1,90	-2,03	-1,80	1
SW403	HDAC6_2039	-1,24	-2,50	-5,23	1
	HDAC6_2040	-2,08	-1,63	0,49	0
	HDAC6_2041	-0,81	-6,58	-4,91	1
	HDAC6_849	-2,00	-2,47	-2,55	1
	HDAC6_850	3,30	2,74	0,83	0
	HDAC6_851	1,99	0,04	-0,27	0
	HDAC6_852	-0,39	-0,95	0,53	0
	HDAC6_853	-0,59	2,01	1,46	0
	HDAC6_854	-2,72	-5,76	-1,64	1
	HDAC6_855	-1,45	0,28	-0,97	0
SW403	PARP1_1147	2,73	-0,23	-2,52	0
	PARP1_1148	-0,95	-2,11	-4,38	1
	PARP1_1149	-1,82	-1,54	-1,95	1
	PARP1_1150	-2,18	1,64	5,08	0
	PARP1_1151	0,07	-1,81	-0,35	0
	PARP1_1152	-2,67	-2,63	-1,70	1
	PARP1_1622	0,39	0,60	1,54	0
	PARP1_2191	3,91	0,53	1,53	0
	PARP1_2192	-5,79	-2,66	-2,95	1

	PARP1_2193	1,76	-2,56	-0,14	0
SW403	PGD_1173	-7,43	-0,27	-2,38	0
	PGD_1174	0,22	-3,31	-1,77	0
	PGD_1175	-8,08	0,81	-5,96	0
	PGD_1176	-5,45	-5,28	-1,29	1
	PGD_1647	0,47	-4,68	-8,65	0
	PGD_1648	-2,76	-3,28	-2,80	1
	PGD_1649	-1,91	-1,62	-4,91	1
	PGD_1819	0,49	0,49	1,09	0
	PGD_1820	-6,62	-3,40	-4,60	1
	PGD_2208	-0,40	0,26	-2,22	0
SW403	PRKCH_1265	2,57	-0,92	-0,75	0
	PRKCH_1266	1,22	0,72	0,34	0
	PRKCH_1611	-0,14	1,27	4,70	0
	PRKCH_1612	-4,34	-0,77	-0,88	0
	PRKCH_2249	-4,00	-3,36	-1,50	1
	PRKCH_2250	4,60	1,36	2,84	0
	PRKCH_2251	-1,55	-1,58	-1,49	1
	PRKCH_2252	-3,27	-2,44	-2,48	1
	PRKCH_2253	-2,14	-0,94	-1,03	1
	PRKCH_2254	8,42	-9,58	-9,63	0
SW403	TUBB_1459	2,75	-2,68	-7,62	0
	TUBB_1460	-10,65	-0,60	-2,37	0
	TUBB_1461	-9,44	-11,47	-2,11	1
	TUBB_2394	-5,05	-3,57	-8,12	1
	TUBB_2395	-3,02	-5,05	-7,67	1
	TUBB_2396	-2,35	-2,50	-2,26	1
	TUBB_2397	0,46	-0,52	-0,38	0
	TUBB_2398	-4,44	-2,47	1,70	0
	TUBB_2399	-2,65	-1,68	-0,69	0
	TUBB_2400	-0,92	-7,40	-1,45	1

Supplementary Table S1C. Expression levels expressed as TMM of each candidate hit in the CRC cell lines used for the synthetic lethal screening.

Gene	CAR1	LIM2099	SW403	WIDR
TUBB	4076.296	10098.391	4903.074	2268.020
EGFR	711.560	387.6250	31.195	280.3801
PGD	2564.576	1263.310	2009.560	1563.810
LAP3	752.781	1085.649	612.888	2131.641
CDK1	302.359	478.156	1635.901	603.011
CDK4	994.588	1089.631	1342.928	895.392
PARP1	968.608	1244.899	926.659	340.057
BIRC5	432.197	1177.517	945.513	245.406
GLS	324.003	955.374	267.322	174.226
PRKAA1	272.887	187.631	214.635	772.220
PRKCI	231.198	147.509	170.540	569.553
MAPK1	192.555	286.090	139.834	190.285
FYN	58.214	334.174	59.743	317.741
HDAC6	225.543	185.989	159.662	296.634
BRAF	74.448	25.649	19.513	187.492
PRKCH	100.0732	12.522	36.291	122.889
HDAC8	51.831	61.586	76.324	60.069

Table S2. Gene Ontology (GO) analysis on candidate hits from the pevonedistat synthetic lethality screening in four CRC cell lines. 17 candidate hits were used to perform a Gene Ontology (GO) analysis. GO biological processes, number of genes, P-values associated to the functional enrichment and gene belonging to each category are reported.

GO Biological Process Term	N. of hits	Hit Gene Count	Genes	Percent of hits that are members of the gene set	Fold Enrichment	*p*-Value
GO:0006468~protein phosphorylation	17	8	MAPK1, BRAF, FYN, PRKCI, PRKCH, PRKAA1, BIRC5, CDK4	47.059	17.329	0.00000010
GO:0043066~negative regulation of apoptotic process	17	6	EGFR, CDK1, BRAF, PRKCI, PRKAA1, BIRC5	35.294	13.025	0.00004874
GO:0008284~positive regulation of cell proliferation	17	5	EGFR, MAPK1, PRKAA1, BIRC5, CDK4	29.412	10.598	0.00081719
GO:0007165~signal transduction	17	5	EGFR, MAPK1, PRKCH, PRKAA1, CDK4	29.412	4.254	0.02115887
GO:0009636~response to toxic substance	17	4	MAPK1, CDK1, CDK4, HDAC6	23.529	46.483	0.00006684
GO:0018105~peptidyl-serine phosphorylation	17	4	MAPK1, CDK1, PRKCI, PRKCH	23.529	31.608	0.00021009
GO:0043524~negative regulation of neuron apoptotic process	17	4	BRAF, FYN, PRKCI, BIRC5	23.529	29.932	0.00024671
GO:0000165~MAPK cascade	17	4	EGFR, MAPK1, BRAF, FYN	23.529	15.080	0.00180911
GO:0051301~cell division	17	4	CDK1, TUBB, BIRC5, CDK4	23.529	11.289	0.00410933
GO:0035556~intracellular signal transduction	17	4	FYN, PRKCI, PRKCH, PRKAA1	23.529	9.804	0.00608930
GO:0070301~cellular response to hydrogen peroxide	17	3	CDK1, PRKAA1, HDAC6	17.647	51.988	0.00131763
GO:0031647~regulation of protein stability	17	3	MAPK1, HDAC8, HDAC6	17.647	42.333	0.00197944
GO:0014066~regulation of phosphatidylinositol 3-kinase signaling	17	3	EGFR, MAPK1, FYN	17.647	37.991	0.00245049
GO:0000187~activation of MAPK activity	17	3	MAPK1, CDK1, PRKAA1	17.647	27.694	0.00455375
GO:0030168~platelet activation	17	3	MAPK1, FYN, PRKCH	17.647	25.768	0.00524030
GO:0000086~G2/M transition of mitotic cell cycle	17	3	CDK1, TUBB, BIRC5	17.647	21.630	0.00735728
GO:0016477~cell migration	17	3	CDK1, FYN, PRKCI	17.647	17.228	0.01139106
GO:0010628~positive regulation of gene expression	17	3	CDK1, BRAF, PRKAA1	17.647	11.310	0.02519840
GO:0042493~response to drug	17	3	CDK1, FYN, CDK4	17.647	9.748	0.03316525
